# CD200 Limits Monopoiesis and Monocyte Recruitment in Atherosclerosis

**DOI:** 10.1161/CIRCRESAHA.119.316062

**Published:** 2021-05-12

**Authors:** Christina Kassiteridi, Jennifer E. Cole, Thibault Griseri, Mika Falck-Hansen, Michael E. Goddard, Anusha N. Seneviratne, Patricia A. Green, Inhye Park, Annelie G. Shami, Tanyaporn Pattarabanjird, Aditi Upadhye, Angela M. Taylor, Ashok Handa, Keith M. Channon, Esther Lutgens, Coleen A. McNamara, Richard O. Williams, Claudia Monaco

**Affiliations:** 1 Kennedy Institute of Rheumatology, Nuffield Department of Orthopaedics, Rheumatology and Musculoskeletal Sciences (C.K., J.E.C., T.G., M.F.-H., M.E.G., A.N.S., P.A.G., I.P., R.O.W., C.A.M.), University of Oxford, UK.; 2 Nuffield Department of Surgical Sciences (A.H.), University of Oxford, UK.; 3 Radcliffe Department of Medicine, RDM Cardiovascular Medicine (K.M.C.), University of Oxford, UK.; 4 Experimental Vascular Biology Division, Department of Medical Biochemistry, Amsterdam UMC, the Netherlands (A.G.S.,).; 5 Institute for Cardiovascular Prevention (IPEK), Ludwig-Maximilians Universität, München, Germany & German Center for Cardiovascular Research (DZHK), partner site Munich Heart Alliance, Munich, Germany (E.L.).; 6 Cardiovascular Research Center, University of Virginia (T.P., A.U., A.M.T., C.A.M.).

**Keywords:** atherosclerosis, bone marrow, inflammation, macrophage, monocyte

## Abstract

Inflammation is a basic component of the pathogenesis of atherosclerosis. CD200 is an immune checkpoint known to control macrophage activation. CD200 recently emerged in the Framingham Heart Study and 2 other cohorts as being potentially relevant in cardiovascular disease. The role of this pathway in cardiovascular disease is unknown.

**Objective:**

We sought to examine the role of CD200 in atherosclerosis.

**Methods and Results:**

Using hypercholesterolemic apolipoprotein-E deficient mice, we demonstrate that whole-body CD200 deficiency augments atherosclerotic lesion formation and vulnerability. Administration of a CD200-Fusion protein reduces neointima formation. Our data show that the CD200-CD200R pathway restrains activation of CD200R+ lesional macrophages, their production of CCR2 ligands, and monocyte recruitment in vitro and in vivo in an air pouch model. Loss of CD200 leads to an excessive accumulation of classical Ly6C^hi^ monocytes and CCR2^+^ macrophages within the atherosclerotic aorta, as assessed by mass cytometry. Moreover, we uncover a previously uncharacterized effect of the CD200/CD200R pathway in limiting dysregulated monopoiesis and Ly6C^hi^ monocytosis in hypercholesterolemic mice. Bone marrow chimera experiments demonstrate that the CD200-CD20R pathway enables 2 complementary and tissue-dependent strategies to limit atherogenesis: CD200 expression by bone marrow–derived cells limits systemic monocytosis, while CD200 expression by nonhematopoietic cells, for example, endothelial cells, prevents local plaque growth. We show that CD200R signaling controls monopoiesis and macrophage activation through inhibiting phosphorylation of STAT1 (signal transducer and activator of transcription 1). Finally, CD200R expression on classical monocytes in peripheral blood of patients with coronary artery disease is associated with a lower burden of coronary artery disease and a more favorable Virtual Histology plaque profile.

**Conclusions:**

The CD200 checkpoint is a key-limiting factor for monopoiesis, monocyte-macrophage activation, and recruitment in atherosclerosis with conserved features in human and mouse. It thus offers a novel potential therapeutic pathway to treat cardiovascular disease.

## Abstract 2

Supplemental Digital Content is available in the text.

**Meet the First Author, see p 218**

Innate immunity is a key component of atherosclerosis, the main cause of cardiovascular disease (CVD). Immune checkpoints are activating or inhibitory receptor-ligand pairs that control activation of T cells and antigen-presenting cells, allowing for tight regulation of immune responses. Immune checkpoints have been shown to play important roles in atherogenesis.^1^

The CD200-CD200R1 ligand-receptor pairing is one such immunoregulatory checkpoint. CD200 (also known as OX-2 or OX-2 membrane glycoprotein) is broadly expressed on a variety of stromal cells and activated lymphocytes,^2^ and it inhibits immune responses by engaging the CD200 inhibitory receptor (CD200R1)^3^ whose expression is restricted to myeloid cells.^3,4^ CD200 contains 2 immunoglobulin superfamily (IgSF) domains, a single transmembrane region and a short cytoplasmic domain and is itself unable to signal downstream. When CD200 interacts with its cognate receptor CD200R1 on myeloid cells, it delivers a selective inhibitory signal.^1^ The CD200-CD200R1 checkpoint is involved in dampening microglial activation in experimental autoimmune encephalomyelitis,^2^ maintaining alveolar macrophage tolerogenic properties during lung infection^5^ and preventing red pulp macrophage expansion.^2^ The effects of the CD200-CD200R1 pathway on monocytes and monopoiesis are unclear as are the mechanisms of increased tissue macrophage numbers in CD200-deficient mice that is seen in a variety of contexts.^2^

Using a system biology approach, Huan et al^6^ identified CD200 as one of the top 10 putative key regulatory genes for changes in peripheral blood gene expression in coronary heart disease. CD200 can be cleaved from cell surfaces and is detectable as soluble CD200 in the blood.^7^ In 2 independent prospective cohort studies, Ganz et al detected CD200 in the peripheral blood of patients with CVD with a targeted proteomics approach with aptamers. Detection of CD200 in the plasma carries a quintile hazard ratio of 1.44 of risk of myocardial infarction, stroke, heart failure, and death.^8^ The significance of the clinical associations described above is poorly understood and the role of the CD200-CD200R pathway in atherogenesis is unknown.

Here, we show that CD200 limits the accumulation of aortic classical monocytes and CCR2+ macrophages during atherogenesis via regulation of monopoiesis and local monocyte recruitment in a tissue-dependent manner, ultimately reducing atherosclerotic plaque progression, inflammation, and necrotic core formation. Our data show that the CD200-CD200R pathway mediates cellular interactions that prevent activation of STAT1 in myeloid cells. CD200R expression is downregulated in human classical monocytes in patients with high coronary artery atherosclerosis burden and its expression is inversely correlated with larger and complex human coronary plaques as assessed by intravascular ultrasound virtual histology. Our data demonstrate strong parallels in the biology and clinical relevance of this pathway in human and mouse, enhancing its translational impact in CVD.

## Methods

### Data Availability

The data, analytic methods, and study materials related to this study are available from the corresponding author upon reasonable request.

### Murine Studies

Mice underwent atherogenesis, bone marrow chimera, and arterial injury studies as described in Methods in the Data Supplement. Murine tissues were harvested and analyzed by immunohistochemistry and immunofluorescent staining, real-time quantitative PCR and flow and mass cytometry, as described in Methods in the Data Supplement.

### Ex Vivo Culture of Cells Isolated From Human Carotid Artery Atherosclerotic Plaques

All patient carotid artery atheroma samples were procured from the Oxford University Hospital trust. The study complied with the Declaration of Helsinki, patients provided written informed consent for use of their tissues and Ethical approval was obtained from National Research Ethics Services and local R&D committee. Single-cell suspensions from fresh diseased intimal arterial segments were obtained, cultured with an agonistic CD200R antibody, and analyzed as described in Methods in the Data Supplement.

### Immunohistochemistry, Human Plaques

Human coronary artery specimens were obtained from autopsy from the Department of Pathology of the Amsterdam Universitair Medische Centra (UMC) and immediately fixed in 10% formalin and processed for paraffin embedding. All use of tissue was in agreement with the Code for Proper Secondary Use of Human Tissue in the Netherlands. Staining was performed as described in Methods in the Data Supplement.

### Mass Cytometry of Human Samples

Twenty subjects, 40 to 80 years old enrolled upon presentation to the Cardiac Catheterization laboratory at the University of Virginia, Charlottesville (UVA), for a medically indicated diagnostic cardiac catheterization were studied. All were outpatients with a stable coronary syndrome. Patient characteristics are in Table I. All study subjects provided written informed consent before enrolment. Protocols and procedures were approved by the Institutional Review Board for human subjects at UVA (IRB No. 15328). Blood sampling and peripheral blood mononuclear cell isolation followed by staining and analysis by cytometry by time of flight was then performed as described in Methods in the Data Supplement.

### Statistical Methods

Data were analyzed with GraphPad Prism (v6.0c, San Diego, CA). To determine if the data had a normal distribution, the Shapiro-Wilk normality test was applied. Normally distributed variables are expressed as mean±SEM, and non-normally distributed variables are expressed as median and interquartile range. Data were analyzed with Student *t* test, Mann-Whitney *U* test, 1-way ANOVA, Kruskal-Wallis test or Spearman correlation as appropriate and as indicated in Table II in the Data Supplement. The significant findings from the planned tests are reported in the Figures.

## Results

### CD200 and CD200R Expression in Atherosclerosis

Expression of CD200 and CD200R during atherogenesis was assessed in aortic roots of chow-fed apolipoprotein E-deficient (*ApoE*^−/−^) mice aged 12, 20, and 28 weeks. CD200 but not CD200R expression significantly increased with time (Figure IA in the Data Supplement). mRNA levels of CD200 in the aorta also increased with age in both wild type (WT) and *ApoE*^−/−^ mice although at 28 weeks, CD200 mRNA was significantly lower in *ApoE*^−/−^ compared with WT mice (Figure IB in the Data Supplement). The cellular expression of CD200 and CD200R in atherosclerotic tissue was then examined. In murine carotid sections, strong CD200 staining along the luminal edge of the lesion correlated with PECAM-1 (CD31) staining and CD200R immunopositivity was associated with regions of CD68 staining (Figure IC in the Data Supplement). Immunofluorescent staining in aortic root sections of *ApoE*^−/−^ mice confirmed endothelial cell CD200 expression and CD200R expression on CD68+ cells (Figure ID and IE in the Data Supplement). Examination of *ApoE*^−/−^ aortas using cytometry by time of flight confirmed that CD200 is abundantly expressed on endothelial cells and is also expressed by smooth muscle cells (Figure IF and IG in the Data Supplement). CD200R expression on aortic myeloid populations was investigated further and was found to be mainly expressed by CD206^+^ macrophage subsets (CD209^+^, and CD209^−^ and CCR2^+^ CD206^int^) and by CD26^−^ cDC2 cells (Figure IH through IJ in the Data Supplement). During atherosclerosis progression, expression of CD200R on aortic monocytes and macrophages significantly increases (Figure IK in the Data Supplement).

CD200-positive endothelial and smooth muscle cells were also evident in human coronary atherosclerotic plaques (Figure IIA in the Data Supplement) while CD200R expression was mainly restricted to myeloid cells (Figure IIB in the Data Supplement). CD200 expression was also assessed in disassociated human carotid plaques by flow cytometry. We found that endothelial cells express high levels of CD200, although it could also be detected on leucocytes, including T cells, B cells and myeloid cells (Figure IIC in the Data Supplement). CD200R expression was mainly restricted to myeloid cells (Figure IIC in the Data Supplement).

### CD200R Activation via Administration of a CD200-Fc Reduces Neointima Formation

Using a well-established model of arterial injury, where lesion formation is accelerated via the placement of a perivascular collar,^9^ the effect of therapeutic provision of CD200 to *ApoE*^−/−^ mice was then assessed. Following collar placement, *ApoE*^−/−^ mice were treated intraperitoneally with a CD200-Fc fusion protein or an IgG1 control, 3× per week for 3 weeks (Figure IIIA in the Data Supplement). Neointima formation, as assessed by the intima:media ratio, was significantly decreased in CD200-Fc fusion protein-treated compared with control-treated mice (0.49±0.1 versus 0.69±0.1; *P*=0.049; Figure IIIB and IIIC in the Data Supplement). No neointima formation was observed in the sham-treated contralateral artery. Lesional collagen content was not significantly different between the 2 treatment groups (Figure IIID and IIIE in the Data Supplement). Surprisingly, lesional macrophage content was significantly increased (Figure IIIF and IIIG in the Data Supplement). CD200-Fc treatment significantly increased the lesional CD206+ area (3.4±0.8% versus 1.5±0.4%, *P*=0.0327; Figure IIIH and IIII in the Data Supplement).

### *Cd200* Deficiency Promotes Atherosclerotic Lesion Development and Vulnerable Plaque Morphology

**Figure 1.**
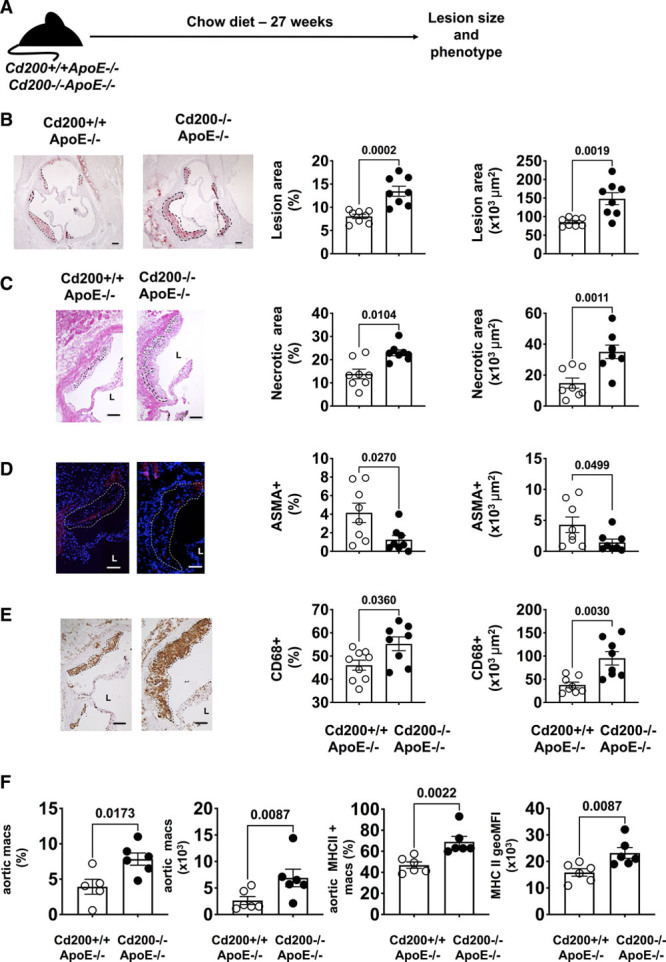
***Cd200* deficiency increases atherogenesis and affects plaque phenotype in 27-wk-old apolipoprotein E-deficient (*ApoE*^−/−^) mice.** **A**, Schematic diagram of the experimental set up. **B**, Representative images of aortic root sections from male *Cd200*^+/+^*ApoE*^−/−^ and *Cd200*^−/−^*ApoE*^−/−^ mice aged 27 wk stained with Oil Red-O and hematoxylin. Dotted lines denote lipid-rich lesion regions of the plaques. Scale bars=100 μm. Graphs show the percentage aortic root lesion area (%, left) and cross-sectional aortic root lesion size (×10^3^ μm^2^, right) (n=8). **C**, Representative images of hematoxylin and eosin (H&E)-stained aortic root sections. Areas of necrosis are denoted by dotted lines. Graphs show percentage (%, left) and absolute (×10^3^ μm^2^, right) and necrotic area in aortic root lesions (n=8). **D**, Representative images of aortic root sections stained with an antibody against smooth muscle cell (SMC) α-actin (Cy3-red) and cell nuclei stained with DAPI (blue) from male *Cd200*^+/+^*ApoE*^−/−^ and *Cd200*^−/−^*ApoE*^−/−^ mice aged 27 wk. Dotted lines highlight lesions. Graphs show aortic root lesion area staining positive (×10^3^ μm^2^ and %) for SMCs (n=8). **E**, Representative images of aortic root sections stained with an antibody against CD68 (brown) and hematoxylin. Graphs show aortic root lesion area staining positive (×10^3^ μm^2^ and %) for CD68 (n=8–9). L=lumen, scale bars=100 μm. Images that best represent the mean of the group are shown. Data points represent the mean of individual mice. Bars show group mean±SEM. **F**, Graphs show the numbers (expressed as percentages and absolute numbers) of arterial F4/80^+^CD68^+^ cells in 27-wk-old *Cd200*^+/+^*ApoE*^−/−^ and *Cd200*^−/−^*ApoE*^−/−^ mice (n=5–6) and the percentage of MHCII^+^ cells and the mean of MHCII expression on macrophages in 27-wk old *Cd200*^+/+^*ApoE*^−/−^ and *Cd200*^−/−^*ApoE*^−/−^ *mice* (n=6). Bars show group median±interquartile range.

Next, the effect of CD200 deficiency was studied in atherosclerosis-prone *ApoE*^−/−^ mice (Figure 1A). *Cd200*^−/−^*ApoE*^−/−^ and *Cd200*^+/+^*ApoE*^−/−^ mice on a chow diet were euthanized at 20 or 27 weeks of age. No statistically significant differences in the serum cholesterol level (Table III in the Data Supplement) or body weight (Table IV in the Data Supplement) were observed between the 2 groups. No significant difference in aortic root lesion size was seen between *Cd200*^+/+^*ApoE*^−/−^ and *Cd200*^−/−^*ApoE*^−/−^ mice at 20 weeks (Figure IVA through IVC in the Data Supplement). However, at 27 weeks of age, *Cd200*^−/−^*ApoE*^−/−^ mice had significantly increased aortic root lesion size compared with *Cd200*^+/+^*ApoE*^−/−^ mice (Figure 1B).

Atherosclerotic plaque phenotype is important for predicting the development of complications of atherosclerosis. The larger aortic root lesion size in *Cd200*^−/−^*ApoE*^−/−^ mice at 27 weeks of age was accompanied by a significant increase in lesional necrotic core area (22.95±1.3% versus 15.1±2.2%, *P*=0.0104; Figure 1C), reduced lesional smooth muscle cell α-actin positive cross-sectional area (1.4±0.5% versus 4.2±1.0%, *P*=0.0270; Figure 1D) and significantly increased lesional macrophage content (53.8±3.0% versus 46.1±2.2%, *P*=0.0360; Figure 1E) but not at the 20-week time-point (Figure IVD through IVI in the Data Supplement).

### *Cd200* Deficiency Affects the Aortic Immune Cell Landscape in *ApoE*^−/−^ Mice

The increase in lesional macrophage content in 27-week-old *Cd200*^−/−^*ApoE*^−/−^ mice was further investigated using multicolor flow cytometry. The gating strategy for live aortic CD45^+^ cells is presented in Figure VA in the Data Supplement. The aortic content of macrophages (gated as in Figure VB in the Data Supplement) in 27-week-old *Cd200*^−/−^*ApoE*^−/−^ mice was double that of *Cd200*^+/+^*ApoE*^−/−^ mice (Figure 1F, Figure VC in the Data Supplement). A significant increase in MHCII (major histocompatibility complex class II) expression by arterial macrophages in 27-week-old *Cd200*^−/−^*ApoE*^−/−^ mice was also observed (Figure 1F). CD200 deficiency led to a small but not statistically significant increase in aortic macrophage content in *ApoE*^−/−^ mice at 20 weeks of age (Figure VD in the Data Supplement).

**Figure 2.**
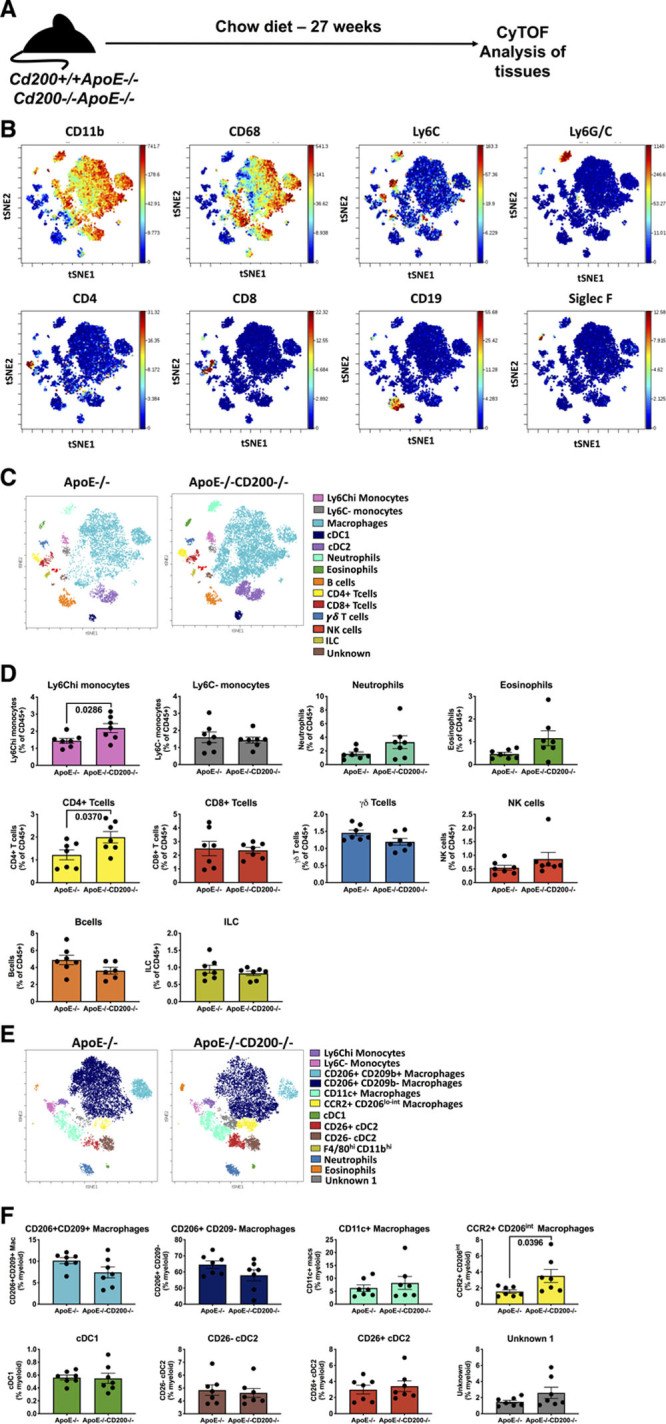
**High-dimensional characterization of leucocyte populations in *Cd200*^+/+^*ApoE*^−/−^ and *Cd200*^−/−^*ApoE*^−/−^ mouse atherosclerotic aortas by mass cytometry.** Single-cell suspensions of aortas from 27- to 30-wk-old *Cd200*^+/+^*ApoE*^−/−^ and *Cd200*^−/−^*ApoE*^−/−^ mice fed a chow diet were stained with a panel of 35 antibodies. For each sample, cells from 2 aortas were pooled. **A**, Schematic diagram of the experimental set up (**B**) viSNE plots of live CD45^+^ cells from a representative *Cd200*^+/+^*ApoE*^−/−^ mouse showing expression of major markers of cell populations. **C**, viSNE plots of live CD45^+^ cells from representative *Cd200*^+/+^*ApoE*^−/−^ and *Cd200*^+/+^*ApoE*^−/−^ mice. The analysis identified 14 populations including myeloid, lymphocyte and unknown subsets. **D**, Bar graphs showing the changes in abundance of the cell populations identified in the viSNE clustering between *Cd200*^+/+^*ApoE*^−/−^ and *Cd200*^+/+^*ApoE*^−/−^ mice. **E**, viSNE plots of myeloid cells (gated as Lin^−^CD11b^lo-hi^) from representative *Cd200*^+/+^*ApoE*^−/−^ and *Cd200*^+/+^*ApoE*^−/−^ mice. The analysis identified 13 subsets. **F**, Bar graphs showing the changes in abundance of the myeloid cell populations identified in the viSNE clustering between *Cd200*^+/+^*ApoE*^−/−^ and *Cd200*^+/+^*ApoE*^−/−^ mice. Data are presented as mean±SEM. Dots represent individual samples (n=7). cDC2 indicates conventional type 2 dendritic cell; CyTOF, cytometry by time of flight; and ILC, innate lymphoid cell.

We next used mass cytometry to more broadly assess how loss of CD200 expression affects immune cell populations during atherogenesis. Single-cell suspensions of aortas from *Cd200*^+/+^*ApoE*^−/−^ and *Cd200*^−/−^*ApoE*^−/−^ mice fed a chow diet were stained with a panel of 35 antibodies (Table V in the Data Supplement) and analyzed as previously described^10^ (Figure 2A). Live CD45^+^ cells were gated, and a viSNE analysis was performed. Major immune cell populations were gated on the basis of marker expression (Figure 2B) and led to 11 immune cell populations being identified (Figure 2C). Significant increases in Ly6C^hi^ monocytes and CD4^+^ T cells were observed in aortas of *Cd200*^−/−^*ApoE*^−/−^ compared with *Cd200*^+/+^*ApoE*^−/−^ mice (Figure 2D). The aortic composition of myeloid cells, gated as Lin^−^CD11b^lo-hi^, was then examined in greater detail using viSNE. Thirteen myeloid cell populations were identified including neutrophils, eosinophils, conventional type 1 dendritic cells (cDC1s), conventional type 2 DCs (cDC2s), Ly6C^−^ and Ly6C^hi^ monocytes and 5 macrophage subsets (Figure 2E). Aortas of *Cd200*^−/−^*ApoE*^−/−^ mice exhibited significantly higher proportions of CCR2^+^CD206^lo^ macrophages than *Cd200*^+/+^*ApoE*^−/−^ aortas (Figure 2F).

### *CD200* Deficiency Promotes Monocyte-Macrophage Recruitment

**Figure 3.**
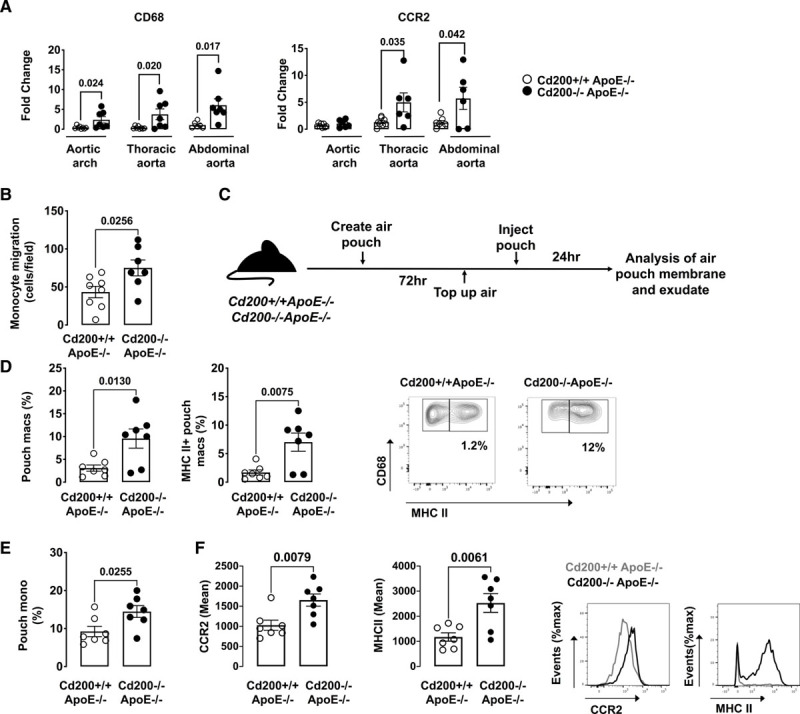
***Cd200* deficiency promotes monocyte-macrophage recruitment.** **A**, Fold change in CD68 (left graph) and CCR2 (C-C chemokine receptor type 2; right graph) gene expression in aortic arch (n=6–8), thoracic aorta (n=6–8), and abdominal aorta (n=6–7) in 27-wk-old *Cd200*^+/+^ *ApoE*^−/−^ and *Cd200*^−/−^ *ApoE*^−/−^ mice. Bars denote group mean±SEM. **B**, Bone marrow (BM) monocytes were isolated from *Cd200*^+/+^*ApoE*^−/−^ and *Cd200*^−/−^*ApoE*^−/−^ mice and Ly6C^hi^ monocyte migration in response to CCL2 was evaluated using a Transwell assay. Graph shows number of migrated cells per field of view (n=7–8). Air pouches were generated on the backs of 15-wk-old *Cd200*^+/+^*ApoE*^−/−^ and *Cd200*^−/−^*ApoE*^−/−^ mice and monocyte recruitment and phenotype evaluated. **C**, schematic diagram of the experimental set up for the in vivo recruitment model. **D**, Graphs and representative contour plots show the numbers of MHCII (major histocompatibility complex class II)+macrophages (gated as live CD45^+^CD11b^+^Ly6G^−^F4/80^hi^CD68^+^ cells) in the air pouch exudate of *Cd200*^+/+^*ApoE*^−/−^ and *Cd200*^−/−^*ApoE*^−/−^ mice (n=7). **E**, Graph shows the numbers of monocytes (gated as live CD45^+^CD11b^+^Ly6G^−^F4/80^int^CD68^+^Ly6C^hi^ cells) in the air pouch membrane of *Cd200*^+/+^*ApoE*^−/−^ and *Cd200*^−/−^*ApoE*^−/−^ mice (n=7). **F**, Graph shows the mean expression of CCR2 and MHCII on Ly6C^hi^ monocytes and histograms show the expression of CCR2 and MHCII in the air pouch membrane monocytes in *Cd200*^+/+^*ApoE*^−/−^ and *Cd200*^−/−^*ApoE*^−/−^ mice. Bars denote group mean±SEM

To elucidate the molecular mechanism of increased lesional macrophage content in *Cd200*^−/−^*ApoE*^−/−^ mice, we quantified expression of inflammatory genes in the aortas of 27-week-old *Cd200*^+/+^*ApoE*^−/−^ and *Cd200*^−/−^*ApoE*^−/−^ mice. *Cd200*^−/−^*ApoE*^−/−^ mice had significantly increased aortic expression of CD68 and CCR2 mRNA compared with CD200 competent mice (Figure 3A). CCR2 and its ligands C-C motif chemokine ligand (CCL) 2 and 7 are crucial for the trafficking of arterial monocytes during atherogenesis.^11–14^ We explored whether *Cd200* deficiency affected the ability of monocytes to migrate in vitro and in vivo. Using an in vitro transwell chamber model, we found that CD200 deficiency promoted CCL2-directed monocyte migration in vitro (Figure 3B). The effect of CD200 on recruitment to tissue was then assessed in a well-characterized air pouch model^15^ (Figure 3C). Compared with *Cd200*^+/+^*ApoE*^−/−^ mice, MHCII^+^ macrophages were significantly increased in the air pouch exudate of *Cd200*^−/−^*ApoE*^−/−^ mice (Figure 3D). Furthermore, there was a significant increase in the recruitment of classical monocytes into the air pouch membrane in CD200-deficient compared with sufficient *ApoE*^−/−^ mice (Figure 3E). The phenotype of these monocytes was also altered in *CD200*^−/−^*ApoE*^−/−^ mice as they expressed significantly higher levels of CCR2 and MHCII (Figure 3F). A significant increase in air pouch membrane neutrophils was also found in *CD200*^−/−^*ApoE*^−/−^ mice (Figure VI in the Data Supplement).

**Figure 4.**
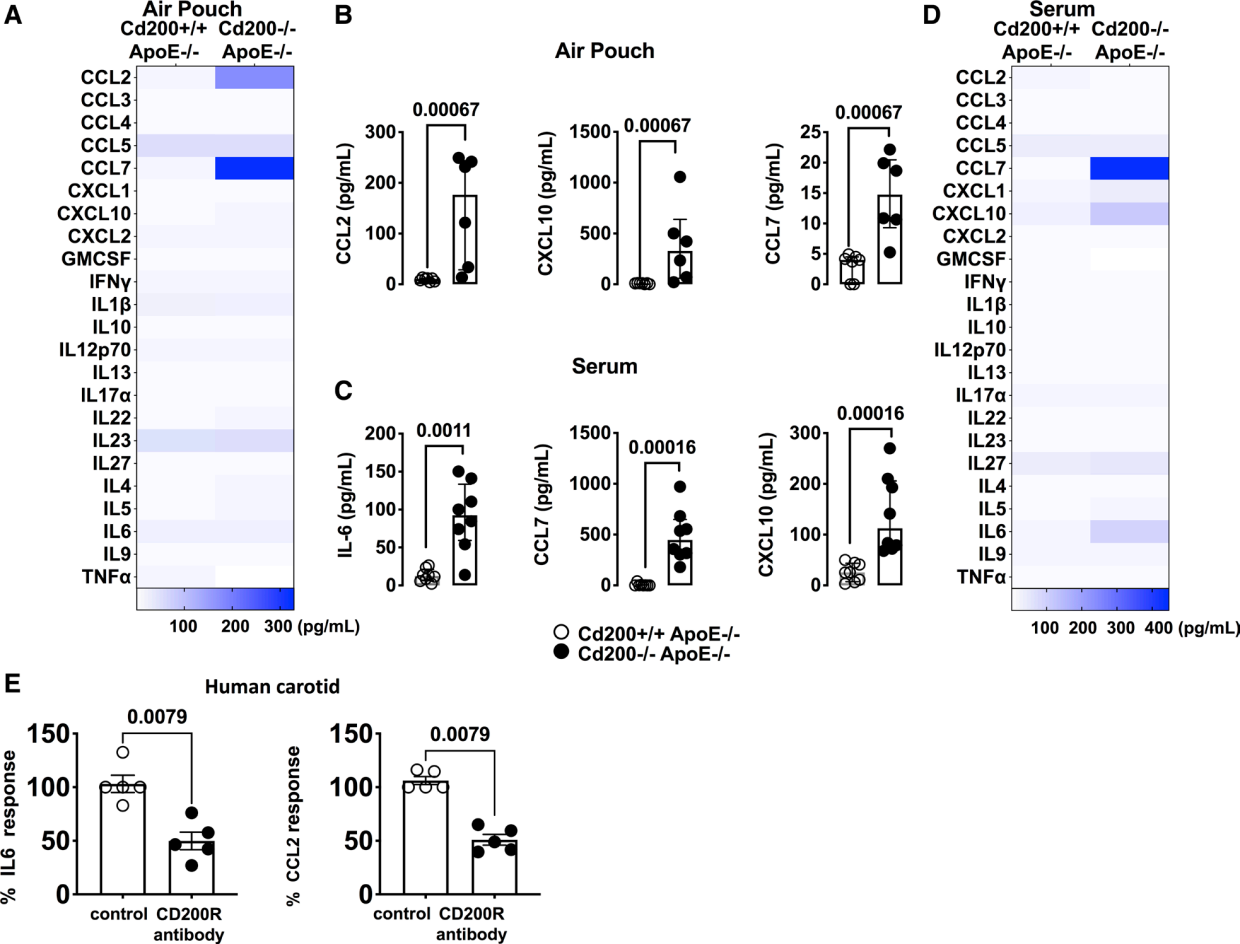
**Increased cytokine/chemokine production in *Cd200*-deficient *ApoE*^−/−^ mice.** **A**, Heatmap shows the expression of cytokines and chemokines in the air pouch exudate of *Cd200*^+/+^*ApoE*^−/−^ and *Cd200*^−/−^*ApoE*^−/−^ mice. **B**, Graphs show CCL2 (C-C motif chemokine ligand 2), CXCL10 (C-X-C motif chemokine 10), and CCL7 production in the air pouch exudate of *Cd200*^+/+^*ApoE*^−/−^ and *Cd200*^−/−^*ApoE*^−/−^ mice (n=6–7). **C**, Graphs show IL6, CXCL10, and CCL7 production in the serum of *Cd200*^+/+^*ApoE*^−/−^ and *Cd200*^−/−^*ApoE*^−/−^ mice. Bars denote group median±interquartile range. **D**, Heatmap shows the expression of cytokines and chemokines in the serum of *Cd200*^+/+^*ApoE*^−/−^ and *Cd200*^−/−^*ApoE*^−/−^ mice (n=8). **E**, Graphs show the percentage (%) of IL (interleukin)-6 (left graph) and CCL2 (right graph) response in human carotid atheroma cells that were either untreated or stimulated with an agonistic CD200R antibody (n=3). Bars denote group median±interquartile range.

Using a multiparametric Luminex analysis measuring 26 cytokines and chemokines, a significant increase in CCL2, CCL7, and CXCL10 (C-X-C motif chemokine 10) expression was evident in the air pouch exudate of *Cd200*^−/−^*ApoE*^−/−^ mice compared with their *Cd200*^+/+^*ApoE*^−/−^ littermates (Figure 4A and 4B). Fittingly, *Cd200*^+/+^*ApoE*^−/−^ and *Cd200*^−/−^*ApoE*^−/−^ mice displayed a significant increase in serum levels of IL (interleukin)-6, CCL7, and CXCL10 (Figure 4C and 4D). This chemokine pattern was consistent with the increase in aortic classical monocytes, CCR2^+^ macs, and CD4 T cells in our mass cytometry data (Figure 2).

Finally, to assess if the CD200-CD200R pathway directly regulated chemokine production, we evaluated the effects of CD200R agonism in human atherosclerosis. We isolated live atheroma cells from human carotids, obtaining a mixed cell type single cell suspension as previously shown.^9^ Cells were then cultured in the presence of an agonistic CD200R antibody or media only. Agonistic CD200R antibody treatment significantly reduced spontaneous IL-6 and CCL2 production in human atheroma ex vivo (Figure 4E). As CD200R is exclusively expressed by myeloid cells in these human atheroma tissues (Figure IIC in the Data Supplement), our data demonstrate that activation of the CD200-CD200R pathway leads to reduction of chemokine production from macrophages in atherosclerosis.

### CD200 Limits Monocytosis and Monopoiesis in *ApoE*^−/−^ Mice

**Figure 5.**
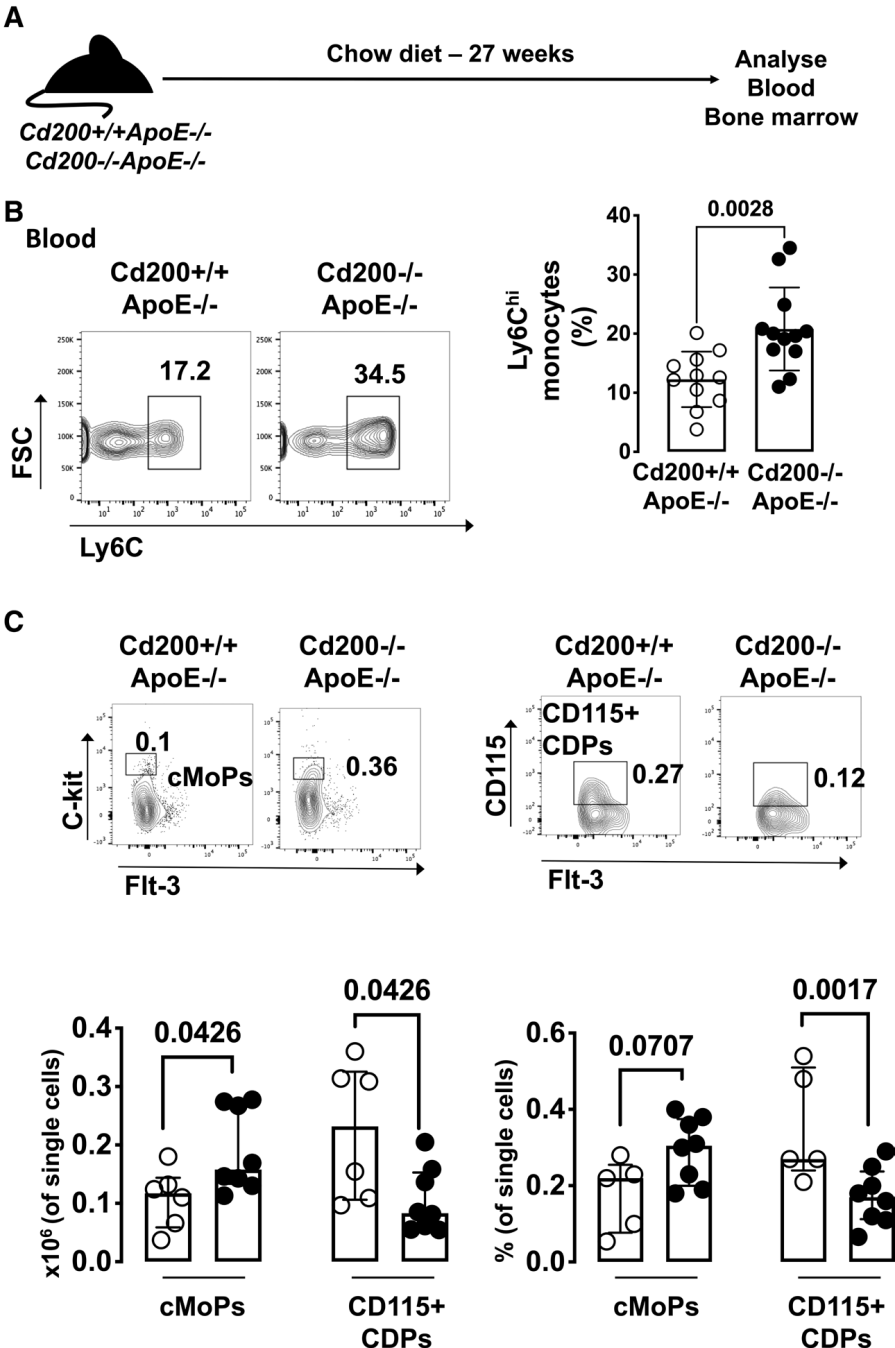
***Cd200* deficiency increases monocytosis and monopoiesis in apolipoprotein E-deficient (*ApoE*^−/−^) mice.** **A**, Schematic diagram of the experimental set up. **B**, Representative contour plots show blood CCR2^+^ monocytes gated on live CD45^+^CD11b^+^Lin^−^Ly6G^−^Ly6C^hi^ cells in *Cd200*^+/+^*ApoE*^−/−^ and *Cd200*^−/−^*ApoE*^−/−^ mice. Graph shows the numbers of live CD45^+^CD11b^+^Lin^−^Ly6G^−^Ly6C^hi^CCR2^+^ inflammatory monocytes (as a % of CD11b^+^cells) in the blood of 27-wk *Cd200*^+/+^*ApoE*^−/−^ and *Cd200*^−/−^*ApoE*^−/−^ mice (n=12). Bars denote mean±SEM. **C**, Contour plots show CD115^+^Ly6C^+^c-kit^+^Flt-3-common monocyte progenitors (cMoPs), and c-kit^−^Flt3^+^CD115^+^ common DC progenitors (CDPs) in 27-wk *Cd200*^+/+^*ApoE*^−/−^ and *Cd200*^−/−^*ApoE*^−/−^ mice. Graph shows the numbers of cMoPs and CD115^+^CDPs (expressed as % of single cells or as absolute numbers) in the BM of 27-wk *Cd200*^+/+^*ApoE*^−/−^ and *Cd200*^−/−^*ApoE*^−/−^ (n=6–8). Bars denote median±interquartile range.

CCR2 is important for the exit of monocytes from the bone marrow (BM) into the circulation.^14^ Using flow cytometry, we assessed whether the increase in aortic monocyte infiltration in CD200-deficient *ApoE*^−/−^ mice was associated with changes in monocyte populations in the blood (Figure 5A). The gating strategy is shown in Figure VIIA in the Data Supplement. Inflammatory Ly6C^hi^ monocytes were significantly increased in the blood of *Cd200*^−/−^*ApoE*^−/−^ compared with *Cd200*^+/+^*ApoE*^−/−^ mice at 27 weeks (Figure 5B) but not at 20 weeks of age (Figure VIIB in the Data Supplement). No significant differences in blood neutrophils or Ly6C^lo^ monocytes were found in 27-week-old *Cd200*^+/+^*ApoE*^−/−^ and *Cd200*^−/−^*ApoE*^−/−^ mice (Figure VIIC and VIID in the Data Supplement).

The role of CD200 deletion on BM-derived macrophage cultures was then evaluated in vitro. Granulocyte-macrophage colony stimulating factor (GMCSF)-generated BM cultures are known to generate a heterogenous population of macrophages and DCs that can be discriminated on the basis of their expression of CD11b and MHCII.^16^ Common monocyte progenitors–derived CD11b^hi^MHCII^int^ macrophages were significantly increased while CD11b^int^MHCII^hi^ DCs, thought to be common DC progenitors-derived, were significantly decreased in *Cd200*^−/−^*ApoE*^−/−^ compared with *Cd200*^+/+^*ApoE*^−/−^ mice (Figure VIIIA in the Data Supplement), suggesting that CD200 signaling may affect GM-CSF-mediated myelopoiesis.

To investigate whether CD200-deficiency dysregulates monopoiesis, we assessed key BM progenitors by flow cytometry. BM progenitors were phenotypically characterized as previously described.^17^ The gating strategy is illustrated in Figure IXA in the Data Supplement. We found a significant increase in both the percentage and numbers of common monocyte progenitors (Figure 5C) in the BM of *Cd200*^−/−^*ApoE*^−/−^ mice, while there was a significant decrease in CD115^+^ CDPs (Figure 5C and Figure IXB in the Data Supplement). This observation was consistent with the changes observed in the GM-CSF-BM cultures.

### CD200R Signaling Limits Monopoiesis via STAT1

**Figure 6.**
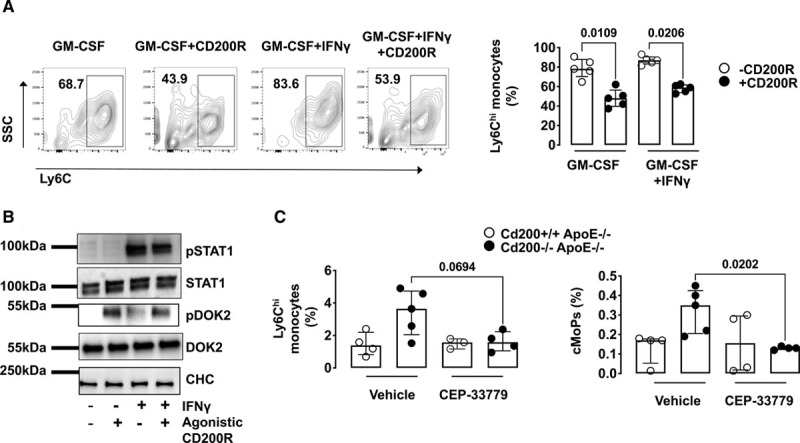
**CD200R agonism inhibits myeloid cell activation via STAT1 (signal transducer and activator of transcription 1).** **A**, Bone marrow (BM) granulocyte-monocyte progenitors (GMPs) were isolated by flow cytometry cell sorting and cultured with GM-CSF in the presence or absence of IFNγ (interferon γ) and/or agonistic CD200R antibody for 4 d. Representative contour plots show Ly6C^hi^ monocytes (gated on CD45^+^CD11b^+^Ly6G-cells) in GM-CSF only, GM-CSF+CD200R antibody, GM-CSF+IFNγ or GM-CSF+IFNγ+CD200R antibody groups. Graphs show the percentage (%) of monocytes within these groups. Three individual experiments were performed (n=5). Bars show median±interquartile range. **B**, Western blot analysis of pSTAT1, STAT1, pDOK2, DOK2, and clathrin heavy chain (CHC) in a macrophage cell line (RAW 264.7) upon stimulation with IFNγ and an agonistic CD200R antibody. Three individual experiments were performed. **C**, Graphs show the percentage of blood monocytes and BM common monocyte progenitors (cMoPs) in *Cd200*^+/+^*ApoE*^−/−^ and *Cd200*^−/−^*ApoE*^−/−^ mice in response to in vivo treatment with a Jak2 inhibitor (CEP-33779) (n=3–5). Bars denote group median±interquartile range. GM-CSF indicates granulocyte-macrophage colony stimulating factor and pDOK2, phosphorylated downstream of tyrosine kinase 2.

Next, we interrogated the effects of CD200R agonism in a model of in vitro monopoiesis. We cultured granulocyte monocyte precursors isolated from *Cd200*^+/+^*ApoE*^−/−^ mice in the presence of GM-CSF with or without IFN (interferon) γ and a CD200R agonistic antibody. The numbers of monocytes generated in these conditions were significantly downregulated in response to CD200R agonistic antibody treatment (Figure 6A).

GM-CSF and IFNγ signaling converge on the transcription factor STAT1.^18^ Thus, we assessed whether macrophage CD200R agonism affected STAT1 phosphorylation in RAW 264.7 cells. Consistent with previous findings,^19^ CD200R signaling activation induced Dok2 phosphorylation (Figure 6B; Figure X in the Data Supplement). Importantly, it also decreased IFN-mediated STAT1 phosphorylation (Figure 6B; Figure X in the Data Supplement).

Jak2 kinase is crucial for the STAT1 phosphorylation that is induced by many stimuli including IFNs. We investigated whether STAT1 inhibition reversed the myeloid phenotype observed in CD200-deficient *ApoE*^−/−^ mice. *Cd200*^−/−^*ApoE*^−/−^ and *Cd200*^+/+^*ApoE*^−/−^ mice were treated orally with 50 mg/kg of a Jak2 inhibitor, CEP-33779, as previously shown.^20^ Decreases in the numbers of BM Common monocyte progenitors and circulating Ly6C^hi^ monocytes were found in *Cd200*^−/−^*ApoE*^−/−^ mice (Figure 6C), suggesting that inhibiting STAT1 phosphorylation reverses the increased supply of monocytes in CD200-deficient *ApoE*^−/−^ mice. Collectively, these findings show that the CD200/CD200R pathway affects the generation of monocytes and activation of macrophages by modulating STAT1 phosphorylation.

### Cell Type–Dependent Effects of CD200 Deficiency on Monocytosis and Atherogenesis

**Figure 7.**
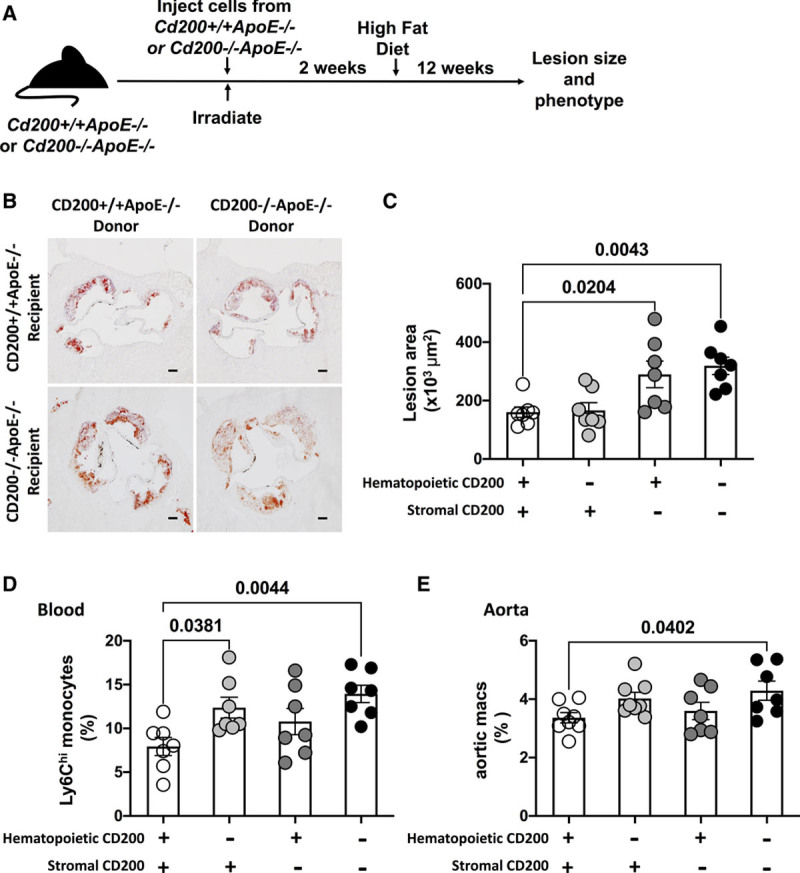
**Stromal *Cd200*-deficiency increases aortic root lesion size in *ApoE*^−/−^ mice.** Male *Cd200*^−/−^*ApoE*^−/−^ *and Cd200*^+/+^*ApoE*^−/−^ littermate mice were sub-lethally irradiated and reconstituted with bone marrow cells from *Cd200*^+/+^*ApoE*^−/−^ *or Cd200*^−/−^*ApoE*^−/−^ mice. **A**, Schematic diagram of the experimental set up. **B**, Representative images of aortic root sections from chimeric mice stained with Oil Red-O and hematoxylin. Images that best represent the mean of the group are shown scale bars=100 μm. **C**, Graph shows the cross-sectional aortic root lesion size (×10^3^ μm^2^, right) (n=7). **D**, Graph shows the numbers of live CD45^+^CD11b^+^Lin^−^Ly6G^−^Ly6C^hi^ inflammatory monocytes (as a % of CD11b^+^ cells) in blood of *Cd200*^+/+^*ApoE*^−/−^ and *Cd200*^−/−^*ApoE*^−/−^ chimeric mice with bone marrow from *Cd200*^+/+^*ApoE*^−/−^ or *Cd200*^−/−^*ApoE*^−/−^ mice (n=7). **E**, Graph shows the number (expressed as percentage) of arterial F4/80+CD68+ cells in *Cd200*^+/+^*ApoE*^−/−^ and *Cd200*^−/−^*ApoE*^−/−^ chimeric mice with bone marrow from *Cd200*^+/+^*ApoE*^−/−^ or *Cd200*^−/−^*ApoE*^−/−^ mice (n=7). Bars denote group mean±SEM, dots represent individual mice.

CD200 can be expressed by stromal or myeloid cells.^2^ To clarify if stromal or hematopoietic CD200 is required for its effects on atherosclerotic lesions and the myeloid compartment, we used a bone marrow chimera approach. *Cd200*^+/+^*ApoE*^−/−^ and *Cd200*^−/−^*ApoE*^−/−^ mice were sub-lethally irradiated and reconstituted with bone marrow cells from *Cd200*^+/+^*ApoE*^−/−^ or *Cd200*^−/−^*ApoE*^−/−^ mice (Figure 7A). Stromal but not hematopoietic CD200 deficiency led to a significant increase in atherosclerotic lesion size (Figure 7B and 7C). However, while hematopoietic CD200-deficiency did not significantly affect aortic root lesion size blood Ly6C^hi^ monocytes were significantly increased (Figure 7D). Although a small increase in aortic macrophage content was observed in the chimeras with hematopoietic CD200 deficiency, only the lack of CD200 in both compartments achieved a statistically significant increase in aortic macrophage content, suggesting that both hematopoietic and stromal sources of CD200 might share in the regulation of aortic macrophage content (Figure 7E).

CD200 and CD200R expression patterns were then examined in the BM and blood of *Cd200*^+/+^*ApoE*^−/−^ mice. In the blood, low CD200 expression was observed in Ly6C^hi^ monocytes and neutrophils (Figure XIA in the Data Supplement) whereas CD200R was found to be highly expressed by Ly6C^hi^ monocytes (Figure XIA in the Data Supplement). Ly6C^hi^ monocytes, common monocyte progenitors and common DC progenitors expressed CD200R in the BM (Figure XIA in the Data Supplement) and expression on these progenitor populations increased with atherosclerosis progression (Figure XIB in the Data Supplement). CD200 was broadly expressed in the BM, with endothelial cells expressing the highest levels of CD200 (Figure XIC in the Data Supplement). CD200 was also expressed in the BM by other stromal cells, lymphoid subsets (in particular B cells and CD4^+^ T cells) and at lower levels by myeloid progenitors (Figure XIA and XIC in the Data Supplement). During atherogenesis, CD200 is downregulated in the vessel wall (Figure IB in the Data Supplement) and on bone marrow endothelial cells (Figure XID in the Data Supplement).

### CD200R Expression in Human Peripheral Blood Is Associated With Lower Coronary Artery Disease

**Figure 8.**
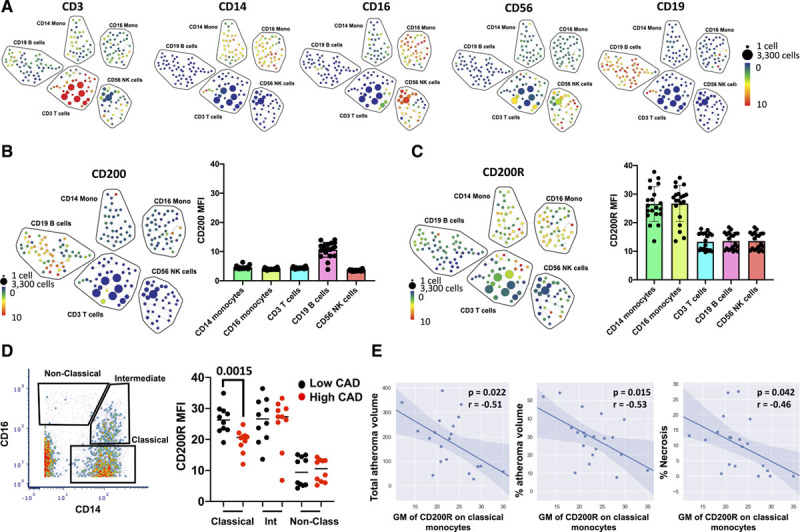
**CD200R expression in human coronary artery disease correlates with disease burden.** **A**, Spanning Tree Progression of Density Normalized Events (SPADE) analysis was performed on CD45^+^ peripheral blood mononuclear cells (PBMCs) obtained from patients with coronary artery disease (CAD) to classify human immune cells into 5 major types including CD3 T cells, CD19 B cells, CD14 monocytes, CD16 monocytes, and CD56 NK cells. **B**, SPADE and graph showing CD200 median expression in cells obtained from patients with CAD. **C**, SPADE and graph showing CD200R median expression in cells obtained from patients with CAD. Key to SPADE tree shows number of cells represented by size of circle and relative expression of the marker across the cells. **D**, Gating of blood monocytes in patients with CAD. Graph shows CD200R median expression within monocyte subsets in high and low CAD patient groups n=10. **E**, Spearman correlation analysis showing inverse association between CD200R expression on CD14 monocytes and atheroma burden and necrosis (n=20). Data were analyzed by using Mann-Whitney Wilcoxon *t* test or spearman correlation. Values are mean±SD.

Multiparameter mass cytometry and high-dimensional analysis were then used to profile CD200 and CD200R expression in circulating immune cell subsets from patients suffering from coronary artery disease (CAD). CAD was characterized by quantitative coronary angiography and Virtual Histology via intravascular ultrasound, as described in the Methods in the Data Supplement. The clinicopathological parameters of the patients are shown in Table I in the Data Supplement. Major immune cell types were identified on the basis of their expression of common cellular markers allowing the identification of 5 immune cell subsets; CD14^+^ monocytes, CD16^+^ monocytes, CD3^+^ T cells, CD19^+^ B cells and CD56^+^ NK cells (Figure 8A). The expression of CD200R and CD200 was then assessed in these populations. CD200 is moderately to highly expressed on B cells and lowly expressed on monocytes (Figure 8B). When CD200 expression was compared between low and high CAD subject groups across these immune subtypes, no significant difference in expression was detected. CD200R was highly expressed on all monocyte subsets (Figure 8C). CD3^−^CD19^−^CD56^−^HLA^−^DR^+^ cells were used to manually gate for classical, intermediate, and nonclassical monocytes to quantify for CD200R level by using mean fluorescent density (Figure 8D). CD200R’s mean fluorescent density on classical (CD14^hi^, CD16^lo^), intermediate (CD14^hi^, CD16^mod^), and nonclassical monocytes (CD14^lo^, CD16^hi^) were compared between subjects with low and high CAD burden as determined by quantitative coronary angiography (as described in the Methods in the Data Supplement).

A significant and selective decrease in CD200R level was observed on classical monocytes in subjects with high CAD burden compared with subjects with a low CAD burden (Figure 8D). CD200Rs’ mean fluorescent density on classical monocytes was also used to correlate with plaque characteristics as assessed via virtual histology (total atheroma volume, % atheroma burden, and % necrosis), and the results suggest significant and moderately high inverse correlations between CD200R level on classical monocytes and total atheroma volume, percentage of atheroma burden and percent necrosis in the plaque (Figure 8E) suggesting that CD200R expression on classical monocytes is associated with a more favorable plaque phenotype.

## Discussion

Monocyte and macrophage supply and recruitment are pivotal events in atherogenesis. CD200 is an inhibitory immune checkpoint known to control macrophage activation through interaction with its cognate receptor CD200R.^2^ We demonstrate that CD200 promotes arterial homeostasis by limiting excessive supply and activation of monocyte-macrophages during atherogenesis via local and systemic cell-dependent mechanisms (Graphical Abstract).

Defective resolution of inflammation is a key driver of atherogenesis^21^ and downregulation of the myeloid lineage is an attractive therapeutic strategy. However, macrophage subsets in atherosclerosis are heterogeneous,^10^ and vascular resident macrophages have homeostatic functions.^22^ Thus, a blanket approach targeting all vascular macrophages could be counterproductive. Classical (Ly6C^hi^) monocytes are the largest subset in mouse blood, and they are the precursors for most lesional macrophages in atherosclerosis.^11,12,23^ Ly6C^lo^ monocytes patrol the endothelium and support vessel wall repair.^24^ The number of circulating monocytes correlates with lesion size in experimental atherosclerosis,^12,13^ and leucocytosis is a risk factor for CVD.^25^ One potential therapeutic strategy for CVD would be to target the supply and activation of monocyte-derived macrophage subsets into and within the arterial wall. CD200 limits atherogenesis by restraining the activation of CD200R+ lesional macrophages and limiting the production of proinflammatory cytokines and CCR2 ligands CCL2 and CCL7 (Figures 3 and 4), thus controlling Ly6C^hi^ monocyte recruitment (Figure 3) and the aortic content of classical Ly6C^hi^ monocytes and CCR2^+^ macrophages (Figures 1 and 2). CD200 also limits the content of aortic CD4 T cells (Figure 2), possibly through reduction of CXCL10 production (Figure 4). Data from the human cohort underscore the importance of this pathway in human classical monocytes and atherosclerosis, by showing that CD200R expression is selectively downregulated in classical monocytes in patients with worse CAD, and is inversely correlated with unfavorable plaque features as assessed by virtual histology (Figure 8). Taken together, our data demonstrate that CD200-CD200R interaction limits macrophage activation and recruitment of classical monocytes in atherogenesis.

Classical (Ly6C^hi^) monocytes develop from granulocyte monocyte progenitor, monocyte dendritic cell progenitor and the recently identified common monocyte progenitor.^17^ Dysregulation of myelopoiesis has been identified as an important contributing factor to atherosclerosis.^12,25^ Hypercholesterolemia is known to cause an increased representation of Ly6C^hi^ monocytes in peripheral blood,^12^ due to dysregulated hematopoiesis. An increase in tissue macrophage numbers in CD200-deficient mice has been observed in a variety of models of inflammation.^2,5^ However, the cellular mechanisms of such increases in myeloid cellularity in organs are unknown. We provide the first evidence that, in a hypercholesterolemic setting, CD200 deletion directly affects the BM causing an increase in monopoiesis to the detriment of the DC lineage. In the steady state, deletion of Dok2 (a known tyrosine downstream of CD200R signaling) has been associated with leukemia and expansion of hematopoietic stem cells.^26^ However, CD200-deficient mice are not characterized by expansion of BM progenitors in the steady state.^27^ This indicates that CD200 deletion per se is not sufficient to induce changes in myelopoiesis. Nevertheless, during hypercholesterolemia and/or inflammation, CD200 deficiency dysregulates myelopoiesis. The main effects of CD200 deletion are evident in chow fed *ApoE*^−/−^ mice at 27 but not 20 weeks of age. This is consistent with the evidence of a blunted age-dependent increase in the expression of CD200 in the murine aorta in advanced atherosclerosis (Figure I in the Data Supplement).

GM-CSF has a crucial role in monocyte generation and macrophage activation in myocardial infarction and inflammatory diseases.^28,29^ IFNγ preferentially induces macrophage formation at the expense of DCs in vitro,^30^ promotes monopoiesis,^31^ and remodels the blood monocyte compartment in gut inflammation by expanding MHCII^+^Sca1^+^Ly6C^hi^ monocytes. STAT1 is a transcription factor hub where several proinflammatory factors converge.^18^ It is phosphorylated on tyrosine residues by Jak kinases and translocates to the nucleus, where it leads to gene activation.^32^ Recently, a clonotypic mutation related to Jak2 function in the BM has been associated with CV risk.^33^ In our study, CD200R1 activation inhibits STAT1 phosphorylation in macrophages, while prevention of STAT1 phosphorylation with a Jak2 inhibitor reversed the excessive monopoiesis in CD200-deficient mice. Thus, CD200 alters the supply and activation of monocyte-macrophages by limiting phosphorylation of STAT1. Our data also suggest that the JAK/STAT pathway might play a part in the systemic monocytosis observed in hypercholesterolemia,^34^ which opens new avenues for exploration of therapeutics.

Expression profiling of CD200 and CD200R indicates that, while CD200R is restricted to myeloid cells, both stromal cells and leukocytes (eg, lymphocytes) express CD200 (Figures I, II, and XI in the Data Supplement). Endothelial cells are the stromal cell type with the highest expression of CD200 (Figure XI in the Data Supplement). Expression profiling of CD200 in the bone marrow shows that among leukocytes, B cells and CD4 T cells express CD200 the most (Figure XI in the Data Supplement). Mass cytometry in patients with coronary artery disease showed that, among blood leukocytes, B cells display the highest expression of CD200 (Figure 8). Bone marrow chimeras demonstrate that hematopoietic deficiency of CD200 fully recapitulates the myeloid phenotype of the Cd200^−/−^ ApoE^−/−^ mice (Figure 7), driving the enhanced blood monocytosis. However, stromal deficiency of CD200 independently phenocopied the lesion size effect of the whole body CD200 deficiency, indicating that the expression of CD200 in stromal cells, such as endothelial cells has a non-redundant role in controlling lesion growth in atherogenesis. The lack of effect of the hematopoietic CD200 deficiency on lesion size suggests that the residual presence of CD200 on stromal cells in the hematopoietic CD200 chimera protects from the increase of plaque burden even in the presence of monocytosis, likely by dampening their recruitment within the vascular compartment. We show that both stromal and hematopoietic CD200 expression contribute to the effect of the CD200-CD200R pathway, each in a distinct and complementary manner at the local and systemic level, respectively. In summary, CD200 has a key role in arterial homeostasis during atherogenesis by limiting excessive monocyte supply and macrophage activation in a tissue-dependent manner.

Our study is not without limitations. The perivascular collar was used as a model of accelerated atherogenesis to study the therapeutic effect of CD200Fc in *ApoE*^−/−^ mice because of limited availability of the gifted compound, which would not have allowed longer atherogenesis studies. Further studies pinpointing the role of the CD200-CD200R pathway in inter-leukocyte and stromal-leukocyte interactions and the mechanisms of intracellular signaling will enhance our understanding of immunomodulatory events in atherogenesis.

In conclusion, our data demonstrate that the CD200 checkpoint exerts a more pervasive control of myeloid functions than previously thought and can act at the level of monocyte supply and recruitment thereby reducing atherosclerosis progression. Our study offers functional insights on the recently emerged clinical association between CD200 and CVD^6,8^ and show conserved features of CD200 biology in human and mouse CVD. Among other immune checkpoints, the CD200/CD200R pathway has the ability to deliver a selective inhibitory signal to monocyte-macrophages that are key cellular culprits in atherosclerosis. Moreover, CD200 deletion in mice reduces lung immunopathology in influenza without significantly affecting viral clearance,^5^ suggesting its activation might be effective without resulting in severe immune suppression. Thus, CD200 is an important immune checkpoint that could offer a novel potential therapeutic avenue for precision targeting of monocyte-macrophages in CVD.

## Sources of Funding

The research leading to these results has received funding from the British Heart Foundation (Grant number PG/19/41/34426); the British Heart Foundation Centre of Research Excellence, Imperial College London; the European Commission under the Seventh Framework Programme (FP7/2007-2013; grant agreement No. HEALTH-F2-2013-602114 [Athero-B-Cell] and grant agreement No. HEALTH-F2-2013-602222 [Athero-Flux]); The Kennedy Trustees, and the Novo Nordisk Foundation (Grant number NNF15CC0018346).

## Disclosures

None.

## Supplemental Materials

Expanded materials and methods

Data Supplement Tables I–VIII

Data Supplement Figures I–XII

References 35–43

## Supplementary Material



## Novelty and Significance

### What Is Known?

- Inflammatory processes, in particular monocyte and macrophage supply and recruitment, are key to the development of atherosclerosis, the main cause of cardiovascular disease
- Activating or inhibitory receptor-ligand pairs (immune checkpoints) can regulate immune responses
- CD200 and its receptor CD200R1 is an inhibitory immune checkpoint that is known to control macrophage activation

### What New Information Does This Article Provide?

- CD200 limits atherosclerotic lesion development in atherosclerosis-prone apolipoprotein E-deficient (*ApoE*^−/−^) mice via regulation of monopoiesis and local monocyte recruitment
- CD200R expression on circulating classical monocytes is inversely correlated with larger and more complex human coronary plaques

Innate immunity is a key component in the pathogenesis of atherosclerosis, the main cause of cardiovascular disease. The immune system can be controlled by immune checkpoints including the CD200-CD200R1 ligand receptor pairing that inhibits macrophage activation. The role of this immune checkpoint in atherosclerosis was unknown. Using atherosclerosis-prone *ApoE*^−/−^ mice with global or tissue-specific CD200 deficiency, we reveal that CD200 plays a protective role in atherogenesis. Loss of CD200 led to development of larger atherosclerotic plaques whereas therapeutic provision of CD200 attenuated neointima formation. Vascular homeostasis is achieved by CD200 via limiting monocyte supply and activation of monocytes/macrophages via both local and systemic cell-dependent mechanisms. In addition, we show that patients with larger and more complex atherosclerotic plaques express lower levels of CD200R on their circulating monocytes. This is the first study to reveal a role for the CD200/CD200R pathway in atherosclerosis and in the regulation of monopoiesis in hypercholesterolemia. These data reveal that the CD200-CD200R1 pathway may be a relevant target for the development of novel therapeutics for the treatment of cardiovascular disease.
