# Supplementary material for: CD200 Limits Monopoiesis and Monocyte Recruitment in Atherosclerosis
Source: Circ Res. 2021 May 12;129(2):280–95. doi: 10.1161/CIRCRESAHA.119.316062 (PMC8260471; doi:10.1161/CIRCRESAHA.119.316062)
Supplement: Supplementary file 1 [file res-129-280-s001.pdf]

Supplemental material

CD200 limits monopoiesis and monocyte recruitment in atherosclerosis

Christina Kassiteridi, PhD[1*], Jennifer E. Cole, PhD[1*], Thibault Griseri [1], Mika Falck-Hansen[1], Michael E. Goddard[1], Anusha Seneviratne[1], Patricia Green[1], Inhye Park[1], Annelie G Shami[4], Tanyaporn Pattarabanjird[5], Aditi Upadhye[5], Angela Taylor[5], Ashok Handa[2], Keith Channon[3], Esther Lutgens[4], Coleen MacNamara[5], Richard O Williams[1], Claudia Monaco[1]

* These two authors contributed equally to the work.

[1]Kennedy Institute of Rheumatology, Nuffield Department of Orthopaedics, Rheumatology and Musculoskeletal Sciences, University of Oxford

[2]Nuffield Department of Surgical Sciences, University of Oxford

[3]Radcliffe Department of Medicine, RDM Cardiovascular Medicine, University of Oxford

[4] Experimental Vascular Biology Division, Department of Medical Biochemistry, Amsterdam UMC

[5] Cardiovascular Research Center, University of Virginia

Address for Correspondence: Claudia Monaco,

Kennedy Institute of Rheumatology,

Roosevelt Drive, Headington, Oxford, United Kingdom

Phone:+44 1865 612636

Fax:+44 1865 612601

Email: claudia.monaco@kennedy.ox.ac.uk

Materials and Methods

Mice

ApoE-/- mice on a C57Bl/6 background were originally purchased from Charles River Laboratories (UK) and bred in-house. *Cd200-/-* embryos on a C57BL/6 background were kindly provided by Merck (New Jersey, USA). *Cd200-/- and ApoE-/-* mice were crossed and littermates were generated, named thereafter as *Cd200+/+ ApoE-/-* and *Cd200-/- ApoE-/-* mice. Where indicated in bone marrow chimaera experiments, some mice had the normal chow diet replaced with a cholate-free high fat diet from Special Diets Services (Essex, UK) consisting of (w/w) cocoa butter (15%), cholesterol (0.25%), maize starch (10%), casein (20%), sucrose (40.5%), cellulose (5.95%), corn oil (1%), 50% choline chloride (2%), methionine (0.2%) and mineral mixture (5.1%). Animals were housed under specific-pathogen free conditions and studied according to UK Home Office regulations and institutional guidelines. Animals were randomly assigned to treatment groups and no animals were excluded from the study.

Power calculations for the number of mice

We made efforts to refine our experiments to reduce the number of animals needed to a minimum. Power calculations were made with G*Power-2 on the basis of our past experience in the detection of changes in atheroma lesion size. The effect size was calculated on the basis of the means and standard deviation from our previous publication with genetic deletion of inflammatory genes. In order to detect an effect size of Cohen's d = 2 with 95% power (alpha = . 05, two-tailed), G*Power suggests we would need a number of 8 data points per group.

Measurement of aortic root lesion development

Male *Cd200-/- ApoE-/-* and *Cd200+/+ ApoE-/-* mice were fed a chow diet up to 20 or 27 weeks of age and then euthanized with a barbiturate overdose and blood collected by cardiac puncture. Hearts were perfused in situ with saline via a cannula inserted into the left ventricle (outflow via an incision in the right atrium) and then frozen in OCT embedding medium. Five-micrometer cryosections were taken of the aortic root for the entire region of the valve leaflets at 100 μm intervals and stained with Oil Red O (ORO) and counterstained with haematoxylin. Images were captured under identical microscope, camera and light conditions. Aortic root sections were coded and analyzed blind. Absolute values for cross-sectional area were obtained by calibrating the software using an image of a micrometer slide taken at the same magnification. The individual lesion areas per aortic root section were averaged to obtain the mean lesion area per mouse. The lesion area fraction was calculated by dividing the mean lesion area by the mean area of the aortic wall and expressed as a percentage. Quantification was performed using Clemex Vision Lite v5.0 (Clemex).

Perivascular collar-induced neointima formation

To accelerate lesion formation, a well-characterised model of arterial injury[9] was used. Female *ApoE-/-* mice aged 22 weeks were anesthetised with isoflurane (4% induction and 2% maintenance) and a non-occlusive collar (length: 2.5 mm; internal bore diameter: 510 μm; Cole-Parmer; Tygon tubing) was placed around the left carotid artery. The right carotid artery was dissected but no cast was placed. Buprenorphine was given as analgesia at the time of surgery. Mice received 10 mg/kg of CD200-Fc fusion protein or isotype control (mouse IgG1 kappa) (provided by Dr Timothy Zeng, Biogen Idec. Inc.) intraperitoneally, three times per week for

three weeks. After three weeks, mice were euthanised with a barbiturate overdose. Following perfusion with saline, both carotid arteries (the left perivascular collar-treated and the right contralateral artery) were dissected, and placed in optimal cutting temperature (OCT) embedding medium, snap-frozen and stored at -80^oC.

For the injured carotid artery, five-micrometer sections were collected distal to the collar. Five sections were collected on each slide and an average of 35 slides were collected. The first 8 alternate slides were stained with Accustain elastic stain kit. Lesion size of the carotid artery (media/intima ratio and percentage of carotid cross-sectional area) was measured following Elastin-Van Gieson staining (Sigma Aldrich).

Bone marrow chimaera generation

Male Cd200+/+ApoE-/- or Cd200-/-ApoE-/- littermate mice aged 8-12 weeks were irradiated twice, 4hr apart with a sublethal dose (5.5Gy) before receiving an intravenous injection of 3-5x10^6 bone marrow cells (from Cd200+/+ApoE-/- or Cd200-/-ApoE-/- donor mice). Mice received antibiotics in their drinking water for 2 weeks. Two weeks post-irradiation, mice were placed on to a cholate-free high fat diet from Special Diets Services (Essex, UK) consisting of (w/w) cocoa butter (15%), cholesterol (0.25%), maize starch (10%), casein (20%), sucrose (40.5%), cellulose (5.95%), corn oil (1%), 50% choline chloride (2%), methionine (0.2%) and mineral mixture (5.1%) for 12 weeks.

H+E staining and Necrosis quantification

H+E staining was performed using a Tissue Tek Prisma/Film automated slide stainer (Sakura, Japan). Slides were fixed in 10% buffered formalin for 10 minutes, rinsed in distilled water and then stained with Harris hematoxylin for 8 minutes, washed in

running tap water for 5 minutes, and differentiated with 0.3% acid alcohol for 2 minutes and rinsed in distilled water. The slides were counterstained with eosin for 2 minutes before being rinsed in distilled water. Necrotic areas were defined as acellular non fibrotic areas exceeding >3000 μm^2 in the aortic root as previously published[16]. Quantification was performed using Clemex Vision Lite v5.0 (Clemex). Absolute values were obtained by calibrating the software using an image of a micrometer slide taken at the same magnification. Necrotic lesion area was calculated by dividing the necrosis area by the lesion area and expressing it as a percentage.

Masson Trichrome staining

Staining for collagen in carotid artery sections was performed using the Masson Trichrome stain kit from TCS biosciences (UK) according to the manufacturer's instructions.

Murine immunohistochemistry and Immunofluorescent staining

Immunohistochemistry was performed using standard ABC protocols as previously published[16]. Bound peroxidase was detected following incubation of the tissues with DAB solution (3, 3'-diaminobenzidine, Vector Laboratories Inc.) and the cell nuclei were counterstained with haematoxylin. Images were captured under identical microscope, camera, and light conditions, coded, and analysed blind. For all primary antibodies used, staining using an appropriate isotype matched control was performed on a consecutive section. For a complete list of antibodies used in this study see Online Table VI. The specificity of the mouse CD200 antibody used for IHC was validated using *CD200-/-ApoE-/-* mice (Online Figure XII).

For immunofluorescence, slides were blocked with 10% goat serum and incubated with primary antibodies listed in Online Table VI, followed by an Alexa Fluor 488- or 568-conjugated anti-IgG secondary antibody or Streptavidin - anti-IgG secondary antibody (Thermo Fisher Scientific) as appropriate. Nuclei were stained with DAPI. Slides were washed in PBS and distilled water before mounting with Prolong mounting medium (Invitrogen). Images of stained sections were taken using an Olympus BX51 microscope.

Murine bone marrow cell cultures

For *in vitro* experiments, bone marrow cells were isolated from *Cd200+/+ApoE-/-* and *Cd200-/-ApoE-/-* mice. The cells were cultured in the presence of RPMI-1640 medium supplemented with 10% heat-inactivated FBS, 100 U/ml penicillin and 100 mg/ml streptomycin in the presence of M-CSF (100 ng/ml) or GM-CSF (20 ng/ml) in 90 mm^2 bacterial dish for 6-8 days and refreshed every two days. Both adherent and non-adherent cells were collected.

***In vitro* myelopoiesis study**

BM cells were harvested as aforementioned and cells were incubated with mouse biotin lineage Ab cocktail (anti-Gr-1, anti-B220, anti-Ter119, anti-CD3e; Invitrogen, Carlsbad, CA, USA), and incubated with Magnisort SAV negative Selection beads (eBiosciences) for 5 minutes. Then, the cells were stained with antibodies against CD34-FITC, c-kit-BV786, Sca-1-PB, CD16/32-APCy7, CD45-Alexa700, and CD11b-BV711. For the lineage negative cocktail, CD4-PerCP-Cy5.5, FcεRI-PerCP-Cy5.5, B220-PerCP-Cy5.5, NKp40-PerCP-Cy5.5 and Streptavidin-PerCP-Cy5.5 antibodies were used (Biolegend). GMPs were gated and then sorted. 3000 cells/well were

cultured in a 12-well plate for 4 days in IMDM medium supplemented with 10% FBS. Cells were cultured with 20 ng/ml SCF, 10 ng/ml GM-CSF (Cell signaling), 10 ng/ml IFNγ (Peprotech, UK) and 10 µg/ml CD200R agonistic antibody (Absolute Antibody, UK).

Flow cytometry

Mice were euthanized and tissues were collected from chow-diet fed *Cd200-/-ApoE-/-* and *Cd200+/+ApoE-/-* mice at 20 or 27 weeks of age or *Cd200+/+ApoE-/-* mice that underwent bone marrow chimaera generation. For bone marrow and citrate-treated blood, erythrocytes were lysed with red blood cell (RBC) lysis buffer (Sigma Aldrich).

Aortas, including the aortic arch, thoracic and abdominal portions were incubated with an enzyme cocktail as previously described[10]. Post-digestion, single cell suspensions were obtained by gently mashing aortas through a 70µm cell strainer. Fc receptors were blocked by incubation with Fc block for 10-20 minutes. Cells were then incubated with primary antibodies (for a complete list of antibodies used in this study see Online Table VI) for 40 minutes at 4°C in the dark and washed twice. Fluorescence minus one (FMO) controls were used as were isotype control antibodies where appropriate/necessary in the set-up of staining protocols and running of samples. Intracellular staining was performed using cell fixation/permeabilization reagents from eBioscience. Cells were additionally stained with the Live/Dead Fixable Dead Cell Stain kit according to the manufacturer's instructions (Invitrogen). Data were acquired using an LSRII or Fortessa flow cytometer (BD) and analysed with FlowJo software (TreeStar).

Mass cytometry of murine aortas

Staining of murine aortas by mass cytometry was performed as described previously [10]. Single cells from two aortas were combined to generate 1 sample. In brief, cells were first stained with a rhodium DNA intercalator (Fluidigm) to identify dead cells followed by barcoding using the palladium-based 20-Plex Pd Barcoding Kit (Fluidigm), according to the manufacturer's instructions. The barcoded samples were then pooled and Fc receptors blocked (BD Biosciences) before staining with metal-conjugated antibodies for 30 min at 4°C. Details of all antibodies are listed in Online Table V. For antibodies that could not be purchased directly conjugated to appropriate metals, conjugation was performed in house using the Maxpar X8 Metal Labelling Kit (Fluidigm) according to manufacturer's instructions. All antibody concentrations were titrated. Following surface antibody staining, the cells were washed and permeabilised using the Foxp3/Transcription Factor Staining Buffer Set (eBiosciences) and stained with antibodies against intracellular markers for 30 min at 4°C. Finally, stained cells were incubated with an Iridium DNA intercalator (Fluidigm) in Maxpar fix and perm buffer (Fluidigm) for up to 48 hours at 4°C. Cells were washed with cell staining buffer (Fluidigm) followed by washing with Maxpar water (Fluidigm). Cells were then filtered through a 40 μm cell strainer before being acquired on a Helios mass cytometer (Fluidigm).

Normalised and debarcoded .fcs files were uploaded to Cytobank (www.cytobank.org) for all gating and further analysis using the automated dimensionality reduction algorithm viSNE[36] that uses the Barnes-Hut implementation of the t-SNE algorithm. After excluding residual normalization beads, doublets, debris and dead cells, samples were gated on CD45+ cells. For myeloid cell viSNE

analysis, cells were gated as Live CD45+Lin$^-$CD11b$^{lo\text{-}hi}$. When running the viSNE analyses, 3000 iterations were run with a perplexity of 80 and theta.

Analysis of murine serum cholesterol

Blood was centrifuged at 6000 rpm for 30 minutes at 4°C and serum collected. Total serum cholesterol levels were measured using Infinity Total Cholesterol (Thermo Scientific), according to the manufacturer's instructions. A calibration serum (Randox Laboratories) with a known cholesterol concentration was used as a reference. The plate was read on a Multiskan plate reader (Thermo Scientific) at 540 and 640 nm wavelengths.

Analysis of murine chemokines and cytokines

Cytokine and chemokine levels were assessed in the serum and supernatant of the airpouch exudate of *Cd200+/+ApoE-/-* and *Cd200-/-ApoE-/-* mice using the 26-plex Mouse ProcartaPlex™ Panel 1 (ThermoFisher Scientific) according to manufacturer's instructions. Samples were read using a Luminex 100 instrument.

Real time quantitative PCR

RNA was extracted from aortas of 27-week old *Cd200-/-ApoE-/-* and *Cd200+/+ ApoE-/- mice* using the Qiagen RNeasy mini kit, according to the manufacturer's instructions. Total RNA was reverse transcribed into cDNA using a High Capacity Reverse Transcription Kit (Life Technologies) according to manufacturer's guidelines. RT-qPCR was performed using TaqMan Gene Expression Assays and TaqMan universal PCR Master Mix (Life technologies) on an ABI 7900HT fast real-time PCR system (Applied Biosystems). PCR amplification was carried out for 40

cycles. Cycling conditions were as follows: UNG incubation, Hold 50°C 2min; polymerase activation, Hold 95°C 10min; 40 Cycles PCR (Deanature 95$_{o}$C 15 sec, Anneal/extend 60°C 60 sec). All samples were analysed in triplicate and were normalized to 18s. The 2-$^{\Delta\Delta Ct}$ method was used to analyse the relative changes in gene expression. A complete list of Taqman assays used in this study is given in Online Table VII.

Transwell assay

Bone marrow (BM) monocytes were isolated using a BM monocyte isolation kit (Miltenyi), according to the manufacturer's instructions. Cells (1x10^{5} cells) were seeded into the upper chambers of the transwells (5 µM pore; Costar) in serum-free media, and allowed to migrate towards 200 ng/ml of MCP-1 (CCL2; Peprotech) in the lower chamber for 2 hours at 37°C. The cells were then fixed with 70% ethanol, and cells that had not migrated were removed from the upper surface of the filter by scraping using cotton swabs. The filters were then stained with Crystal Violet stain (Sigma-Aldrich) for 20 minutes, washed in PBS and mounted onto coverslip dishes, and observed by light microscopy. The number of cells that had migrated across the filters was determined by counting the number of cells in at least 5 random fields (images acquired with a ×20 objective). The migrated cells in the bottom well were used for flow cytometry to confirm efficient isolation of monocytes.

Air pouch recruitment model

Briefly, air pouches were formed on the backs of male *Cd200-/-ApoE-/-* and *Cd200+/+ApoE-/-* mice according to standard protocols[15]. Twenty-four hours post-injection, mice were culled and the air pouches were lavaged with 3 mL PBS (the air

pouch exudate). Cytokines were measured in the supernatants of pouch exudates by Luminex. The air pouch membrane was enzymatically digested in 1 mL of RPMI containing 10 µg DNAse and 2.5 µg Liberase (Sigma). Leucocytes in the air pouch wall or fluid were assessed by flow cytometry.

Western Blotting

RAW 264.7 (mouse macrophage) cells were pelleted and lysed in IP buffer complemented with a protease and phosphatase inhibitor cocktail (Thermofisher). The samples were then assayed for protein content, diluted in sample loading buffer and heated for 5 min at 95 °C. Proteins were separated by 4-10% standard SDS–polyacrylamide gel electrophoresis. The proteins were transferred electrophoretically onto a PVDF membrane using Trans-Blot Turbo Transfer system (Bio-RAD). Blots were probed with polyclonal antibodies against STAT1, pSTAT1, dok-2, and pdok-2 (Cell signaling) overnight at 4°C. Clathrin (BD Biosciences) was used as an endogenous control. Bands were visualised by chemiluminescence.

JAK inhibition

In selected experiments a Jak2 inhibitor, CEP-33779 (Selleckchem) was administered orally in suspension form, twice a day, in a dimethyl sulfoxide (DMSO) (1% final concentration), PEG400 and Tween-80 vehicle at 50 mg/kg (three times a week) in 27-week-old *Cd200-/-ApoE-/-* and *Cd200+/+ApoE-/-* mice.

Ex vivo culture of cells isolated from human carotid artery atherosclerotic plaques

Fresh diseased intimal arterial segments were dissected from carotid endarterectomy specimens under a dissecting microscope. Single cell suspensions were obtained by enzymatic digestion[41]. Freshly isolated atheroma cells were cultured at 1×10^6 cells/ml in RPMI containing 10% FBS (Biosera, Ringmer, UK) in at least three replicate wells. Cytotoxicity was assessed using an MTS Assay kit (Abcam) according to manufacturer's instructions. Cells were incubated with 10 µg/ml of a commercially-available, well-published[35] agonistic CD200R antibody (Absolute Antibody) overnight then supernatants were removed and IL-6 and CCL2 expression were assessed by ELISA (eBiosciences).

Immunohistochemistry, human plaques

Prior to staining human coronary artery sections were deparaffinised and dehydrated in a graded series of ethanol. In preparation for CD200 and CD200R immunodetection, endogenous peroxidase was quenched by incubation in 0.3% H2O2 in methanol. Heat-mediated antigen retrieval using sodium citrate buffer (Vector Laboratories Inc., Burlingtame, CA, USA) at pH 6 was then performed by boiling the sections for 15 min and unspecific binding was blocked by incubation with 10% bovine serum albumin (Sigma Aldrich, St. Louis, MO, USA) for 30 min. Sections were then incubated with antibodies detecting either CD200 (1:250; ab203887, Abcam, Cambridge, UK) or CD200R (1:100; ab198010, Abcam) for 1 h after which the Mach3 Rb HRP-Polymer detection kit (M3R531 H, Biocare Medical, Elkridge, MD, USA) was used according to the manufacturer's instructions. As negative controls, staining was performed omitting the primary antibodies. Positive

immunoreactivity was visualized with 3,3'diaminobenzidine (DAB; DAKO/Agilent, Santa Clara, CA, USA) and counterstaining was performed using Mayer's haematoxylin (Sigma-Aldrich). Slides were mounted with Entellan (Merck, Darmstadt, Germany).

For double-staining of human coronary artery sections, endogenous biotin was blocked using an Avidin/Biotin blocking kit (Vector Laboratories) according to the manufacturer's instructions and unspecific binding prevented with 4% foetal calf serum (30min). Heat-mediated antigen retrieval was performed at 100°C, using either sodium citrate buffer at pH 6 (CD200, CD200r, CD68, smooth muscle α-actin) or TE antigen retrieval buffer at pH 9 (CD31) before incubation with both the first and second primary antibody. Human coronary artery sections were incubated with primary antibodies against CD200, CD200r, CD68 (1:300, ab201340, Abcam), smooth muscle α-actin (α-SMA; 1:3000; F3777, Sigma Aldrich), or CD31 (1:20; M082301-2, DAKO/Agilent, Santa Clara, CA, USA), followed by incubation with secondary biotinylated antibodies recognizing rabbit (CD200 and CD20R; 1:400, DAKO/Agilent), mouse (CD68 and CD31; 1:500, Jackson ImmunoResearch, Cambridgeshire, UK) or FITC-conjugated (α-SMA, 1:600, Jackson ImmunoResearch) antibodies. The alkaline phosphatase standard Vectastain ABC kit (Vector Laboratories) was then used according to the manufacturer's instructions. ImmPact Vector Red or Vector Blue chromogens (Vector Laboratories) were used to visualize positive immunoreactivity. Slides were mounted with Vectamount AQ mounting medium (Vector Laboratories).

Images were recorded with a Leica DM6000 microscope with Las 4.1 software (Leica Microsystems, Wetzlar, Germany).

Mass cytometry of human samples

20 subjects, 40-80 years old enrolled upon presentation to the Cardiac Catheterization laboratory at the University of Virginia, Charlottesville (UVA), for a medically indicated diagnostic cardiac catheterization were studied. All were outpatients with a stable coronary syndrome. Patient characteristics are in Online Table I. Patients were excluded if they had any of the following: any acute illness, type 1 diabetes, current acute coronary syndrome (ACS), autoimmune disease or on immunosuppressive therapy, prior organ transplantation, anemia, pregnancy, HIV infection, or no vessel suitable for intravascular ultrasound (IVUS). All study subjects provided written informed consent prior to enrolment. Protocols and procedures were approved by the Institutional Review Board for human subjects at UVA (IRB# 15328).

Intravascular ultrasound (IVUS) acquisition

IVUS was performed on the least angulated vessel (either left anterior descending artery, circumflex artery or right coronary artery) providing the longest length for evaluation in accordance with the standards of the American College of Cardiology for image acquisition on a non-infarct related artery[40]. 50 U/kg of intravenous heparin and 150 µg of intracoronary nitroglycerin were given prior to advancement of a 2.6 F, 30 MHz IVUS catheter (Volcano Corporation) over a guidewire into the target vessel. The transducer was positioned distally and a motorized pullback was performed at 0.5 mm/sec with images being obtained at 30 frames/sec for a minimum of 30 mm.

The R-100 pullback device (Volcano Corporation) is a non-disposable reusable pullback device specifically designed for use in clinical trials. Each device is individually calibrated and inspected to meet the required accuracy of ± 0.03 mm for pullback speeds of 0.5 mm/sec. Digital images were stored for analysis

Virtual histology and atheroma burden analysis

Plaque composition was assessed using VH. Grayscale images obtained by ultrasound backscatter were used to define the outer circumference of the vessel wall and then the lumen manually by two blinded investigators according to the guidelines for acquisition and analysis of IVUS images by the American College of Cardiology[40]. Every 60th image was analyzed at 1 mm intervals for a total of 40 mm along the length of the artery beginning at the proximal end. Following manual editing of contours, VIAS (Volcano Image Analysis Software) was utilized to reconstruct plaque tissue composition with four identifiable color-coded major components (i) dark green: fibrous plaque that consists of densely packed collagen (ii) light green: fibro-fatty plaque comprised of collagen and interspersed lipid (iii) red: necrotic core that includes cholesterol clefts, foam cells, and micro-calcifications and (iv) white: calcified plaque without adjacent necrosis[42]. Plaque components were expressed as a percentage of total intima area. Interobserver and intraobserver correlation coefficients for our laboratory were 0.96 and 0.98, respectively. Atheroma burden was measured for the 40 mm segment. For segments that are prohibitive to analysis secondary to calcium, the closest usable segment was substituted.

Atheroma burden and stenosis were calculated as below,

$$\text{Atheroma Burden: } \frac{\Sigma \text{ atheroma CSA}}{\Sigma \text{ EEM CSA}} \times 100$$

(CSA= cross-sectional area, EEM= external elastic membrane)

$$\text{Stenosis: } \frac{\text{Vessel Area-Lumen Area}}{\text{Vessel Area}} \times 100$$

Quantitative Coronary Angiography (QCA)

Patients underwent standard cardiac catheterization with two orthogonal views of the right coronary artery and four of the left coronary artery according to accepted standards. QCA was performed using automatic edge detection at an end diastolic frame. For each lesion, the frame was selected based on demonstration of the most severe stenosis with minimal foreshortening and branch overlap. Computer software was used to calculate the minimum lumen diameter, reference diameter, percent diameter stenosis, and stenosis length. Analysis was performed by blinded, experienced investigators. The Gensini score was used to assign a score of disease burden to each patient[39,43]. Briefly, each artery segment is assigned a score of 0-32 based on the percent stenosis. The severity score for each segment was multiplied by 0.5 – 5, depending on the location of the stenosis. Scores for all segments were then added together to given a final score of angiographic disease burden. Score adjustment for collateral was not performed for this study[39,43]. Patients with Gensini score higher than 32 were classified as high coronary artery disease (CAD) burden, and patients with Gensini score lower or equal to 32 were classified as low CAD burden.

Blood sampling and peripheral blood mononuclear cell isolation

Arterial blood was drawn just before IVUS and collected in BD vacutainer K2 EDTA tubes containing 5.4 mg EDTA and processed at room temperature (RT) within two hours of collection. First, whole blood in vacutainers was centrifuged at 400 x g for 10 min at RT to remove platelet-rich plasma. Then, volume was made up by adding PBS containing 5% fetal bovine serum (FBS) and blood was further diluted 1:1 with PBS/FBS. Peripheral blood mononuclear cells (PBMCs) were separated from granulocytes and red blood cells by Ficoll-Paque density-gradient centrifugation (Ficoll-Paque PLUS, GE Healthcare Biosciences AB) and SepMate-50 (Stemcell Technologies Inc.) following the manufacturer's protocol. A small aliquot was used for cell counting and trypan blue staining was performed to distinguish live and dead cells. PBMCs were immediately cryopreserved in freezing solution (90% FBS/10% DMSO; 2.5-5 x 106 cells/tube) using Mr. Frosty (Thermo Fisher Scientific) according to manufacturer's protocol and stored in liquid nitrogen until used for experiments.

CyTOF antibody panel and sample staining

All metal-conjugated antibodies were purchased from Fluidigm and purified unlabelled antibodies from the vendors as shown in Online Table VIII. Purified unlabelled antibodies were conjugated in-house using the MaxPAR antibody labeling kit (Fluidigm) according to manufacturer's protocol. After determining the percent yield by measurement of absorbance at 280 nm, the metal-labeled antibodies were diluted in Candor PBS Antibody Stabilization solution (Candor Bioscience GmbH) for long-term storage at 4°C.

Frozen PBMCs samples were thawed and washed twice with warm complete media (RPMI supplemented with 5% FBS, 1 mM sodium pyruvate and Pen-Strep) and then

rested at 37°C for one hour. All PBMC samples had viability in the range of 85-98%. 1 x 10^6 cells per subject were used for CyTOF staining. All 20 samples were individually stained for 20 minutes at RT with some metal conjugated surface marker antibodies, prior to barcoding as some epitopes were found to be sensitive to paraformaldehyde fixation. Samples were multiplexed using the palladium-based 20-Plex Pd Barcoding Kit (Fluidigm) according to the manufacturer's protocol. Barcoded samples were then combined into a single tube prior to Fc receptor blocking (Biolegend) and staining with a cocktail of the balance of the remaining metal-conjugated antibodies for 30 minutes at RT with frequent gentle shaking (total 34 antibodies). Cells were washed with Maxpar Cell Staining Buffer (CSB) (Fluidigm) and then incubated with Iridium DNA intercalator (Fluidigm) in Maxpar Fix and Perm Buffer (Fluidigm) for 20 minutes at RT. Prior to acquisition, cells were washed once with CSB followed by two washes with Cell Acquisition Solution (Fluidigm) and filtered through a 35 µm nylon mesh (Corning) just before being acquired on a Helios mass cytometer (Fluidigm). EQ Four Element Calibration Beads (Fluidigm) were added as per the manufacturer's directions prior to acquisition.

CyTOF data pre-processing and subsequent analysis

Data obtained from the Helios instrument were in .fcs file format. EQ Four Element Calibration Beads (Fluidigm) were used for data normalization by using bead intensities as described[37]. Data was normalized using the Nolan lab MATLAB normalizer available free on github.com (http://github.com/nolanlab/bead-normalization/releases). The updated debarcoding tool developed by the Zunder's lab[38] available free on github.com was used to debarcode samples (https://github.com/zunderlab/single-cell-debarcoder). This single-cell debarcoding

software tool provides normalized barcode separation distance and mahalanobis distance as parameters for every sample, which allows for sample-specific precision filtering by manual gating on these debarcode parameters in FlowJo or Cytobank. Normalized and debarcoded .fcs files were then further analyzed in FlowJo version 10.3 for Mac (FlowJo LLC, Ashland OR), or uploaded to Cytobank for all gating and subsequent SPADE analysis (https://github.com/nolanlab/spade). To ensure accurate sample identity prior to analysis, sample-specific stringency adjustments by manual 2-D plotting on normalized barcode separation distance and mahalonobis distance was performed. Gating on positive Iridium staining (^{191}Ir DNA intercalator) identified intact cells. Doublets (events high in Iridium) were excluded as well as debris (events that were negative for Iridium). Samples were then gated on CD45+ 89Y. Immune cell clusters generated by SPADE analysis in Cytobank were exported and mean/median expression values for markers were used to plot graphs. All markers except CD45 were used as the clustering channels for the SPADE analysis.

Online Tables

**Variable**<br>Count [%] or Median [± SD]	**CAD Low**<br>(n=10)	**CAD High**<br>(n=10)	**P-value**
**General Characteristics**			
Age (Years)	61 [±7.77]	60 [±10.54]	0.88
% Female	60%	60%	
Race (Caucasian)	8 [80%]	9 [90%]	0.59
Ethnicity (Non-Hispanic)	8 [80%]	8 [80%]	
Diabetes (Yes)	2 [20%]	3 [30%]	0.61
Current Smoker (Yes)	1 [10%]	1 [10%]	
BMI	32 [±6.10]	31 [±4.73]	0.85
Tri	98.7 [±49.5]	106.2 [±45.9]	0.74
HDL	51.3 [±12.4]	43.7 [±20.6]	0.36
LDL	98.7 [±22.6]	98.0 [±32.1]	0.96
**Medications**			
Diuretics (Yes)	2 [20%]	4 [40%]	0.33
Beta Blockers (Yes)	5 [50%]	4 [40%]	0.65
Calcium Channel Blockers (Yes)	2 [20%]	3 [30%]	0.61
Angiotensin converting enzyme (Yes)	2 [20%]	3 [30%]	0.61
Angiotensin II Receptors (Yes)	1 [10%]	1 [10%]	
NSAID (Yes)	7 [70%]	8 [80%]	0.61
**Disease Severity**			
Gensini Scores	1.8 [±2.70]	53.4 [±15.89]	0.001

Online Table I. Characteristics of patients with a stable coronary syndrome. The Man-Whitney U test was used to describe differences between the two groups for continuous variables. The Chi-Square test was used to describe differences between the two groups for categorical variables.

Figure		Test
Figure 1	B-E (Lesion size and phenotyping)	Student’s t Test (unpaired) with Bonferroni-Dunn method multiple test correction
	F	Mann-Whitney Test (unpaired)
Figure 2	D, F – CyTOF analysis	Student’s t Test (unpaired)
Figure 3	A-F	Student’s t Test (unpaired)
Figure 4	B-C	Mann-Whitney Test (unpaired) with Bonferroni-Dunn method multiple test correction
	E	Mann-Whitney Test (unpaired)
Figure 5	B	Student’s t Test (unpaired)
	C	Mann-Whitney Test (unpaired)
Figure 6		Mann-Whitney Test (unpaired)
Figure 7		One-way ANOVA with Dunnett’s multiple comparisons test
Figure 8	D	Mann-Whitney Test
	E	Spearman correlation
Online Figure I	A,G and K	Kruskal-Wallis with Dunn’s multiple comparisons test
	B	Mann-Whitney Test (unpaired) with Bonferroni-Dunn method multiple test correction
Online Figure III		Student’s t Test (unpaired)
Online Figure IV		Student’s t Test (unpaired) with Bonferroni-Dunn method multiple test correction
Online Figure V		Student’s t Test (unpaired)
Online Figure VI		Student’s t Test (unpaired)
Online Figure VII	B-C	Mann-Whitney Test (unpaired)
	D	Student’s t Test (unpaired)
Online Figure VIII		Mann-Whitney Test (unpaired)
Online Figure IX	B	Mann-Whitney Test (unpaired) with Bonferroni-Dunn method multiple test correction
Online Figure X		Kruskal-Wallis with Dunn’s multiple comparisons test
Online Figure XI	B, D	Kruskal-Wallis with Dunn’s multiple comparisons test

Online Table II. Statistical tests used in the analysis of the data in each figure.

**Mouse genotype**	**Age(wk)**	**Serum cholesterol ± SEM (mg/dL)**	**p vs. *Cd200+/+ApoE-/-***
Cd200+/+ApoE-/-	27	473.1±23.55	
Cd200-/-ApoE-/-	27	464.4±62.13	p = 0.89

Online Table III. Serum cholesterol levels in 27 week old, chow fed *Cd200+/+ApoE-/-* and *Cd200-/-ApoE-/-* mice; n=7-8.

**Mouse genotype**	**Age (weeks)**	**Final body weight ±SEM (g)**	**p vs. *Cd200+/+ApoE-/-***
Cd200+/+ApoE-/-	27	33.9 ± 0.3	
Cd200-/-ApoE-/-	27	32.8 ± 0.6	p=0.17
Cd200+/+ApoE-/-	20	32.8 ± 0.6	
Cd200-/-ApoE-/-	20	31.6 ± 0.9	p=

Online Table IV. Body weights of *Cd200+/+ApoE-/-* and *Cd200-/-ApoE-/-* mice. n=8-14.

Metal Label	Antigen	Clone	Supplier	Catalogue Number	Conjugation
141 Pr	Ly-6G/C (Gr-1)	RB6-8C5	Fluidigm	3141005B	Fluidigm
142 Nd	CD11c	N418	Fluidigm	3142003B	Fluidigm
143 Nd	IL7Ra	A7R34	Biolegend	135029	In-house
144 Nd	XCR1	ZET	Biolegend	148202	In-house
145 Nd	TCRgd	GL3	Biolegend	118101	In-house
146 Nd	CX3CR1	SA011F11	Biolegend	149002	In-house
147 Sm	CD45	30-F11	Fluidigm	3147003B	Fluidigm
148 Nd	CD11b (Mac-1)	M1/70	Fluidigm	3148003B	Fluidigm
149 Sm	CD19	6D5	Fluidigm	3149002B	Fluidigm
150 Nd	CD24	M1/69	Fluidigm	3150009B	Fluidigm
151 Eu	CD64	X54-5/7.1	Fluidigm	3151012B	Fluidigm
152 Sm	CD3e	145-2C11	Fluidigm	3152004B	Fluidigm
153 Eu	CD200R	OX-110	Biolegend	123902	In-house
154 Sm	CD43	S11	Biolegend	143202	In-house
155 Gd	Lyve-1	ALY7	eBioscience	14-0443-95	In-house
156 Gd	CD169	3D6.112	Biolegend	142402	In-house
158 Gd	CD206	C068C2	Biolegend	141702	In-house
159 Tb	F4/80	BM8	Fluidigm	3159009B	Fluidigm
160 Gd	CD26	DPP-4	Biolegend	137802	In-house
161 Dy	CD103	2E7	Biolegend	121402	In-house
162 Dy	Ly6C	HK1.4	Fluidigm	3162014B	Fluidigm
163 Dy	CCR2	SA203G11	Biolegend	Custom order	In-house
164 Dy	CD172a (SIRPa)	P84	Biolegend	144002	In-house
165 Ho	CD161 (NK1.1)	PK136	Fluidigm	3165018B	Fluidigm
166 Er	CD209b (SIGNR1)	2C7B27	Biolegend	147802	In-house
167 Er	SIGLECF	E50-2440	BD	552125	In-house
168 Er	CD8a	53-6.7	Fluidigm	3168003B	Fluidigm
169 Tm	TCRb	H57-597	Fluidigm	3169002B	Fluidigm
170 Er	CD90.2	30-H12	Biolegend	105333	In-house
171 Yb	CD44	IM7	Fluidigm	3171003B	Fluidigm
172 Yb	CD4	RM4-5	Fluidigm	3172003B	Fluidigm
173 Yb	SIGLECH	551	Biolegend	129602	In-house
174 Yb	I-A/I-E (MHCII)	M5/114.15.2	Fluidigm	3174003B	Fluidigm
175 Lu	CD68	FA-11	Biolegend	137002	In-house
176 Yb	CD45R (B220)	RA3-6B2	Fluidigm	3176002B	Fluidigm

Online Table V. Antibodies used for murine CyTOF. CyTOF=cytometry by time-of-flight.

Antibody	Clone	Use	Source	Catalogue Number
B220 PerCP-Cy5.5	RA3-6B2	FC	Biolegend	103236
CCR2 PE	SA203G11	FC	Biolegend	150610
CCR2 APC	475301	FC	R&D systems	FAB5538A
CD117 (c-kit) BV785	2B8	FC	BD Biosciences	564012
CD11b BV711	M1/70	FC	Biolegend	101242
CD11b PE	M1/70	FC	eBioscience	12-0112-81
CD11b PerCP	M1/70	FC	Biolegend	101230
CD11b PerCP-Cy5.5	M1/70	FC	Biolegend	101228
CD11c PerCP-Cy5.5	N418	FC	Biolegend	117328
CD11c APC-Cy7	N418	FC	Biolegend	117324
CD135 (Flt3) APC	A2F10	FC	Biolegend	135310
CD115 PE	AFS98	FC	Biolegend	135506
CD115 APC	AFS98	FC	Biolegend	135510
CD135 (Flt3) PE	A2F10	FC	Biolegend	135306
CD150 BV605	TC15-12F12.2	FC	Biolegend	115927
CD16/32 APC-Cy7	2.4G2	FC	BD Biosciences	560541
CD19 PerCP	6D5	FC	Biolegend	115532
CD200	OX-90	IHC	AbD Serotec	MCA1958
CD200 APC	OX-90	FC	Biolegend	123810
CD200R	OX-110	IHC	AbD Serotec	MCA2281
CD200R PE	OX-110	FC	Biolegend	123908
CD200R APC	OX-110	FC	Biolegend	123916
CD200R Fitc	OX-110	FC	Biolegend	123910
CD206 APC	C068C2	FC	Biolegend	141708
CD206 Biotin	C068C2	IF	AbD Serotec	MCA2235B
CD3 FITC	145-2C11	FC	BD Bioscience	553062
CD3 PerCP-Cy5.5	145-2C11	FC	BD Bioscience	551183
CD31 (PECAM-1)	Polyclonal	IF	Abcam	ab28364
CD31 (PECAM-1) BV605	390	FC	Biolegend	102427
CD31 (PECAM-1) APC-Fire 750	390	FC	Biolegend	102434
CD34 FITC	RAM34	FC	eBiosciences	11-031-82
CD4 BV421	RM4-5	FC	Biolegend	100543
CD4 PerCP-Cy5.5	RM4-5	FC	eBioscience	45-0042-82
CD45 Alexa700	30-F11	FC	Biolegend	103128

CD45 APC	30-F11	FC	Biolegend	103111
CD45 APC-Cy7	30-F11	FC	Biolegend	103116
CD45 BV421	30-F11	FC	Biolegend	103134
CD45 BV605	30-F11	FC	Biolegend	103139
CD45 BV711	30-F11	FC	Biolegend	103147
CD45 FITC	30-F11	FC	Biolegend	103108
CD45 PE	30-F11	FC	Biolegend	103106
CD45 PECy7	30-F11	FC	Biolegend	103113
CD45 PerCP	30-F11	FC	Biolegend	103130
CD45 PerCP-Cy5.5	30-F11	FC	Biolegend	103132
CD51 PE	RMV-7	FC	Biolegend	104105
CD64 APC	X54-5/7.1	FC	Biolegend	139306
CD68	FA-11	IHC/IF	AbD Serotec	MCA1957GA
CD68-AF647	FA-11	IF	Abd Serotec	MCA1957A647
CD68 PECy7	FA-11	FC	Biolegend	137016
CD68 PerCp Cy5.5	FA-11	FC	Biolegend	137010
CD8 BV711	53-6.7	FC	Biolegend	100759
CD86 FITC	PO-3	FC	Biolegend	105110
DC-SIGN (CD209) PE	LWCO6	FC	eBioscience	12-2091-82
F4/80 BV605	BM8	FC	Biolegend	123133
F4/80 PE	BM8	FC	Biolegend	123110
FcεRIα PerCP-Cy5.5	MAR-1	FC	Biolegend	134320
Isotype control (Rabbit IgG)	Polyclonal	IHC/IF/FC	Abcam	ab37415
Isotype control (Rat IgG1)	4639	IHC/IF/FC	AbD Serotec	MCA6004GA
Isotype control (Rat IgG2α κ)	R35-95	IHC/IF/FC	AbD Serotec	MCA1212
Ki-67 BV421	B56	FC	BD Bioscience	562899
Ly6C PECy7	HK1.4	FC	Biolegend	128018
Ly6G APC	1A8	FC	Biolegend	127614
Ly-6G/C (Gr-1) PE	RB6-8C5	FC	Biolegend	108408
MerTK PE	108928	FC	R&D systems	FAB5912P
MHCII BV421	M5/114.15.2	FC	Biolegend	107632
MHCII FITC	M5/114.15.2	FC	Biolegend	107606
NKp46 PerCP-Cy5.5	29A1.4	FC	Biolegend	137610
PDGFRa PE-Dazzle	APA5	FC	Biolegend	135921
Sca-1 PerCp Cy5.5	D7	FC	Biolegend	108123

Sca-1 Pacific Blue	D7	FC	Biolegend	108120
SMC α-actin	1A4	IF	Sigma Aldrich	A5691-.2ml
TER-119 PE	TER-119	FC	Biolegend	116207
TER-119 PerCP-Cy5.5	TER-119	FC	Biolegend	116228

Online Table VI. Antibodies used for IHC, IF and FC. IHC = Immunohistochemistry, IF = Immunofluorescence/Confocal Microscopy, FC = Flow Cytometry.

Gene name	Taqman assay
CCL2	Mm00441242_m1
CCR2	Mm99999051_gh
IFNγ	Mm01168134_m1
CD200	Mm00487740_m1
CD200R1	Mm00491164_m1
CD68	Mm03047340_m1
IL-12β	Mm01288993_m1

Online Table VII. Taqman assays used in experiments.

Metal Label	Antigen	Clone	Supplier	Catalogue Number
89 Y	CD45	HI30	Fluidigm	3089003B
141 Pr	CD70	Polyclonal	Biolegend	
142 Nd	CD19	HIB19	Fluidigm	3142001B
143 Nd	CD123	6H6	Fluidigm	3143014B
144 Nd	CD11b	ICRF44	Fluidigm	3144001B
146 Nd	IgD	IA62	Fluidigm	3146005B
147 Sm	CD11c	BU15	Fluidigm	3147008B
148 Nd	PD-L1 (CD274)	29E.2A3	Fluidigm	3148017B
149 Sm	CD200	OX-104	Fluidigm	3149007B
150 Nd	CD43	84-3C1	Fluidigm	3150006B
151 Eu	CD14	M5E2	Fluidigm	3151009B
152 Sm	CD95	DX2	Fluidigm	3152017B
153Eu	TIM-3	F38-2E2	Fluidigm	3153008B
155 Gd	BAFFR (CD268)	11C1	Biolegend	316902
156 Gd	CD86	IT2.2	Fluidigm	3156008B
158Gd	CD137L	5F4	Fluidigm	3158022B
159 Tb	CD22	HIB22	Fluidigm	3159005B
160 Gd	CD200R	OX-108	Biolegend	329302
162 Dy	CD80	2D10.4	Fluidigm	3162010B
163 Dy	CD95L	NOK-1	Biolegend	306402
164 Dy	GITRL	109114	R+D Systems	MAB6942-100
165 Ho	CD40	5C3	Fluidigm	3165005B
166 Er	Siglec 10	Polyclonal	Biolegend	
167 Er	CD27	L128	Fluidigm	3167006B
168 Er	OX40L	159403	R+D Systems	MAB10541-100
169 Tm	CD24	ML5	Fluidigm	3169004B
170 Er	CD3	UCHT1	Fluidigm	3170001B
171 Yb	CD20	2H7	Fluidigm	3171012B
172 Yb	IgM	MHM-88	Fluidigm	3151026D
173 Yb	CD137	4B4-1	Fluidigm	3173015B
174 Yb	HLA-DR	L243	Fluidigm	3174001B
175 Lu	PD-1	EH12.2H7	Fluidigm	3175008B
176 Yb	CD56	N901	Fluidigm	3176009B
209 Bi	CD16	3G8	Fluidigm	3209002B

Online Table VIII. Antibodies used for human CyTOF. CyTOF=cytometry by time-of-flight.

Online Figures

Online Figure I

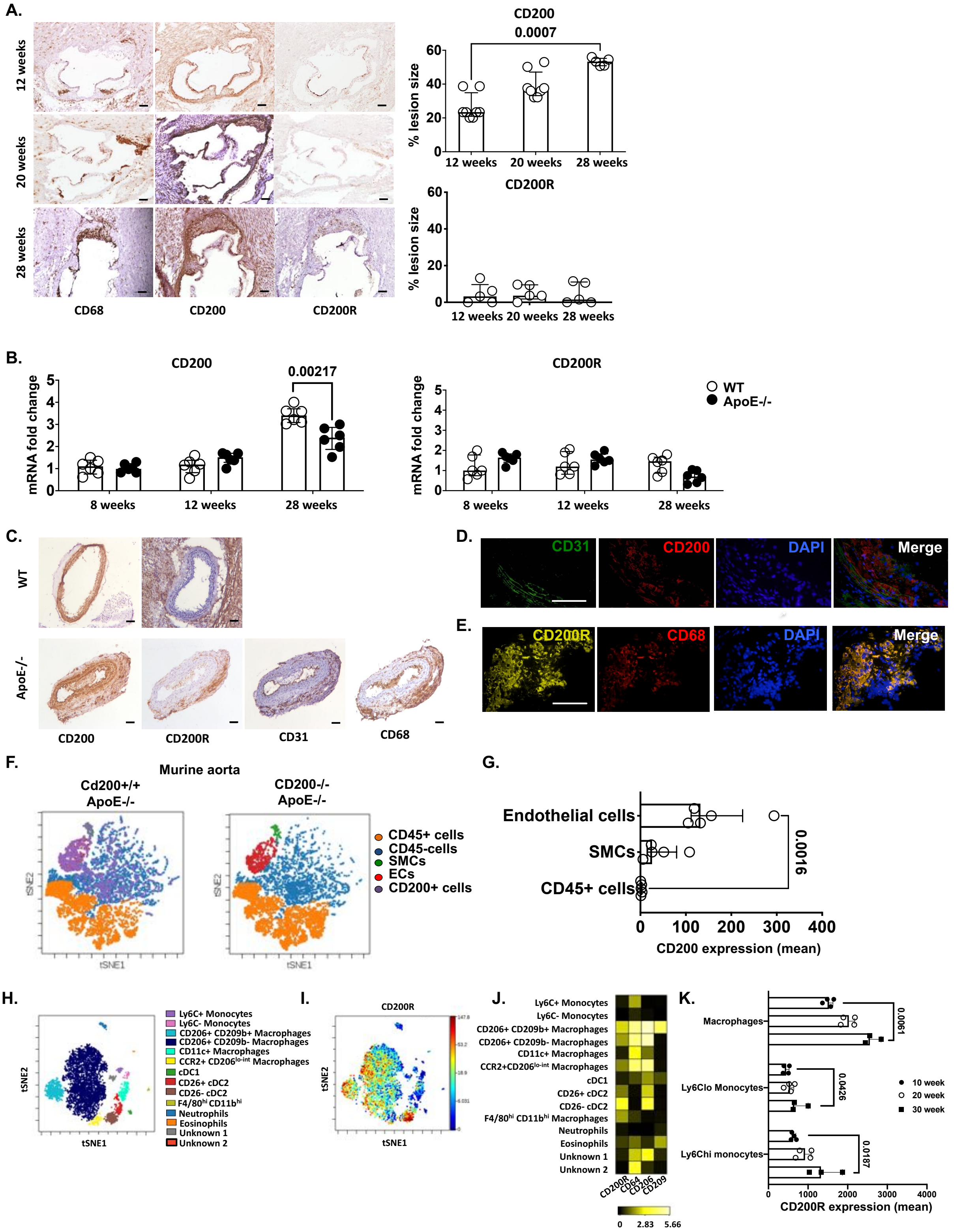

Online Figure I. CD200 and CD200R expression in wild-type and atherosclerotic mice. (A) Representative images of CD200, CD200R and CD68 staining in aortic root cross-sections from chow-fed 12, 20 and 28 week old apolipoprotein E-deficient (ApoE-/-) mice. Images that best represent the mean of the group are shown. Graphs show the protein levels of CD200 and CD200R expression (as a percentage of the cross-sectional lesion area) in aortic roots from 12, 20 and 28 week old ApoE-/- mice (n=5-8). (B) mRNA levels of CD200 (left graph) and CD200R (right graph) in aortas of 8,12 and 28 week old wild type (WT) and ApoE-/- mice (n=6). (C) Representative images of carotid sections from WT and ApoE-/- mice stained with antibodies against CD200, CD31, CD200R and CD68. Images that best represent the staining pattern observed are shown. Scale bars = 100µm (D) Immunofluorescent staining of CD200 (red), PECAM-1 (CD31; green) and DAPI (blue) in the aortic roots of 20-week old *ApoE-/-* mice. (E) CD200R (yellow) and CD68 (red) staining in the aortic roots of 20-week old of ApoE-/- mice. DAPI (blue) stains cell nuclei. Scale bars = 100µm. (F) viSNE analysis of CD200 expression in the aortas of ApoE-/- mice by mass cytometry. (G) Graph shows mean CD200 expression in cells from the aortas of ApoE-/- mice (n=5). (H) viSNE viSNE plots of myeloid cells (gated as Lin-CD11b$^{lo\text{-}hi}$) from representative *Cd200+/+ApoE-/-* mice. (I) viSNE plot of myeloid cells from a representative *Cd200+/+ApoE-/-* mouse showing expression of CD200R. (J) heatmap of expression of CD200R, CD64, CD206 and CD209 in myeloid cells of a representative *Cd200+/+ApoE-/-* mouse. (K) Graph shows expression of CD200R on aortic monocytes and macrophages in *Cd200+/+ApoE-/-* mice aged 10, 20 and 30 weeks. Bars denote group median ± interquartile range.

Online Figure II

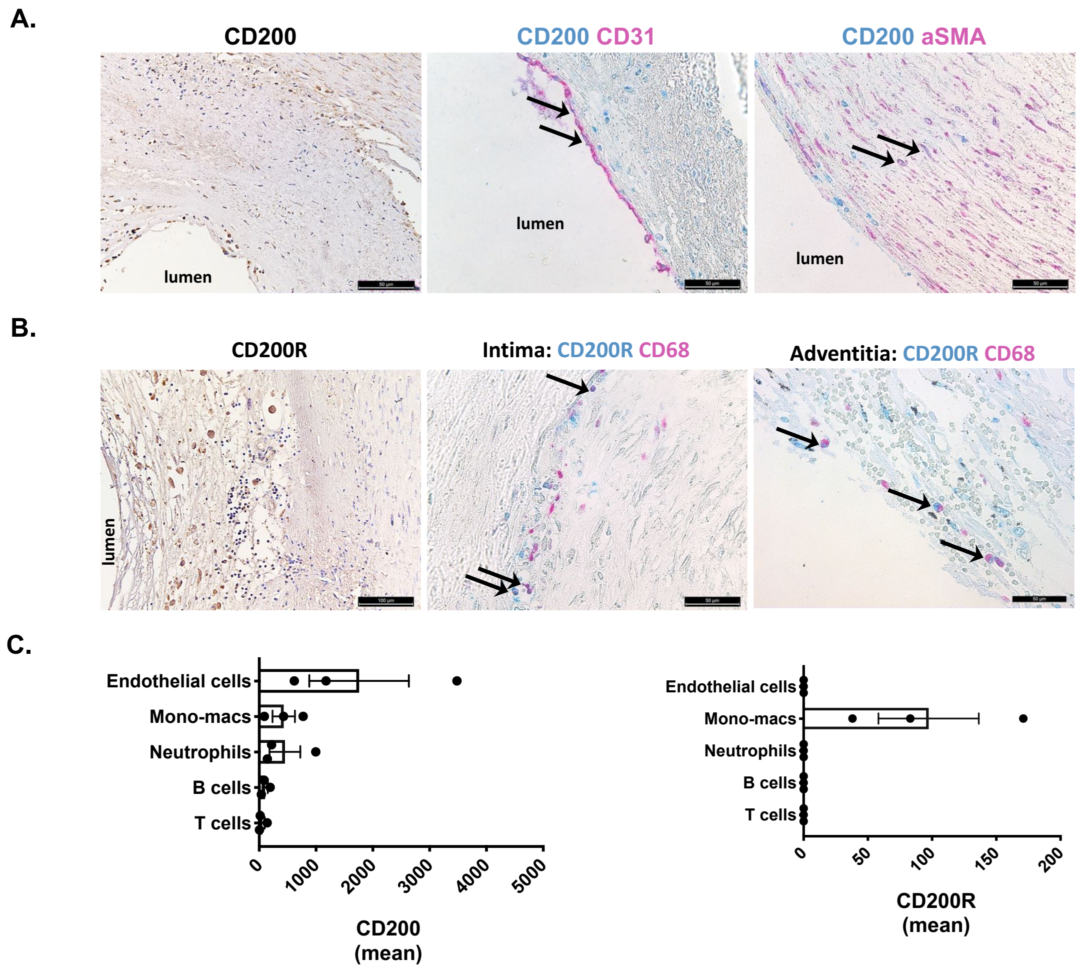

Online Figure II. CD200 and CD200R expression in human atherosclerosis Representative images of human coronary plaques stained with (A) an antibody against CD200 (left) or co-stained with antibodies against CD200 (blue)/CD31 (red) (middle) or CD200 (blue)/α smooth muscle actin (SMA)(red) (right) and (B) an antibody against CD200R (left) or co-stained with antibodies against CD200R (blue)/CD68 (red) in the intima (middle) or in the adventitia (right). Images that best represent the staining pattern observed are shown. Arrows indicate double positive cells. Scale bar = 50µm. (C) Graphs show the mean CD200 expression in endothelial cells, neutrophils, monocytes, T cells and B cells from human carotid artery cells isolated *ex vivo* (n=3) and the mean CD200R expression in CD45+ and CD45- cells in human atheroma cells (n=3). Bars denote group median ± interquartile range.

Online Figure III

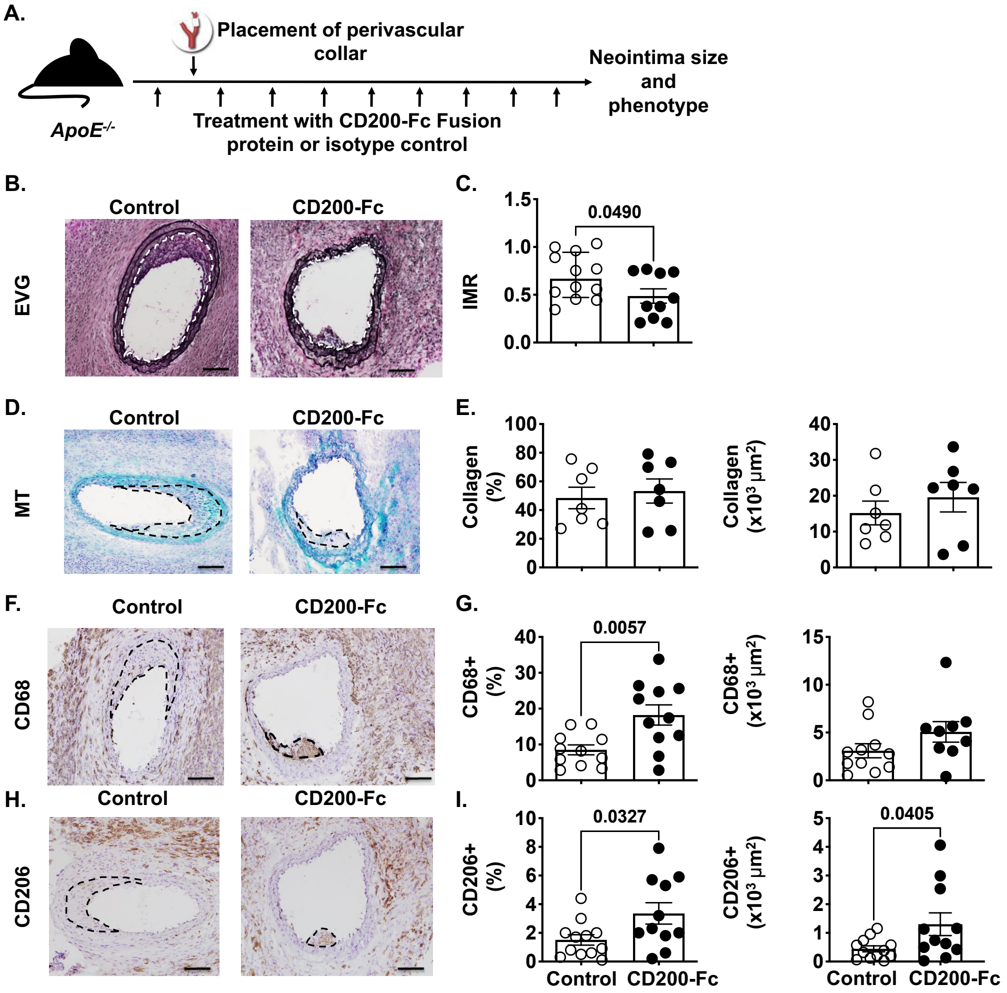

Online Figure III. CD200R ligation exerts atheroprotective effects. Apolipoprotein E-deficient (ApoE-/-) mice with a perivascular collar on the carotid artery were treated with a CD200-Fc fusion protein or IgG control (control). Mice were culled 21 days after collar placement. (A) schematic diagram of the experimental set up (B) Representative images of injured carotid artery sections stained with elastin Van Gieson (EVG). (C) Graph shows intima:media ratio (IMR) of carotid arteries 21 days after injury (n=10-12). (D) Representative Masson's Trichrome (MT) staining of injured carotid arteries. (E) Graphs show the percentage and absolute lesion area staining positive for Masson's Trichrome in IgG (control) or CD200-Fc-treated carotids (n=7). (F) Representative images of injured carotid arteries stained with an antibody against CD68 and haematoxylin. (G) Graphs show the percentage and absolute lesion area staining positive for CD68 in IgG (control) or CD200-Fc-treated injured carotids (n=11). (H) Representative images of injured carotid arteries stained with an antibody against CD206 and haematoxylin. (I) Graphs show the percentage and absolute lesion area staining positive for CD206 in IgG (control) or CD200-Fc-treated carotids (n=11-12). Images that best represent the mean of the group are shown. Dotted lines show internal elastic lamina (IEL). Scale bars = 100µm. Data points represent the mean of individual mice. Horizontal line denotes group mean ± SEM.

Online Figure IV

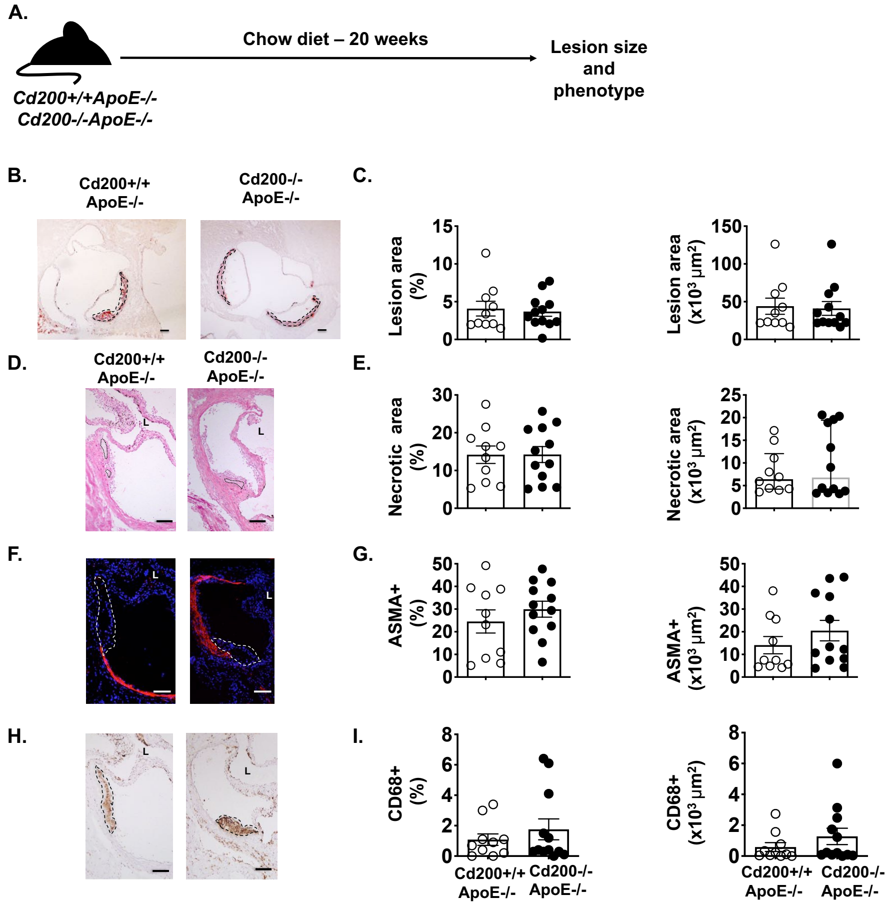

Online Figure IV. Lesion phenotype in 20-week old CD200+/+ApoE-/- and CD200-/-ApoE-/- mice. (A) schematic diagram of the experimental set up (B) Representative images of aortic root sections from male *Cd200+/+ApoE-/-* and *Cd200-/-ApoE-/-* mice aged 20 weeks of age stained with Oil Red-O and haematoxylin. Dotted lines denote lipid-rich lesion regions of the plaques. Scale bars = 100µm. (C) Graphs show the percentage aortic root lesion area (%, left) and cross-sectional aortic root lesion size (x10^{3} µm^{2}, right) (n=10-12) (D) Representative images of haematoxylin and eosin (H&E)-stained aortic root sections. Areas of necrosis are denoted by dotted lines. (E) Graphs show percentage (%, left) and absolute (x10^{3} µm^{2}, right) necrotic area in aortic root lesions. (n=7-10). (F) Representative images of aortic root sections stained with an antibody against smooth muscle cell (SMC) α-actin (Cy3-red) and cell nuclei stained with DAPI (blue) from male *Cd200+/+ApoE-/-* and *Cd200-/-ApoE-/-* mice aged 20 weeks. Dotted lines highlight lesions. (G) Graphs show aortic root lesion area staining positive (x10^{3}µm^{2} and %) for SMCs. (n=8,12). (H) Representative images of aortic root sections from male *Cd200+/+ApoE-/-* and *Cd200-/-ApoE-/-* mice aged 20 weeks stained with an antibody against CD68 (brown) and haematoxylin. Dotted lines highlight lesions (I) Graphs show lesion area staining positive (x10^{3}µm^{2} and %) for CD68 (n=8-12). Images that best represent the mean of the group are shown. L = lumen, scale bars = 100 µm. Individual data points represent the mean of individual mice. Horizontal line denotes group mean ± SEM.

Online Figure V

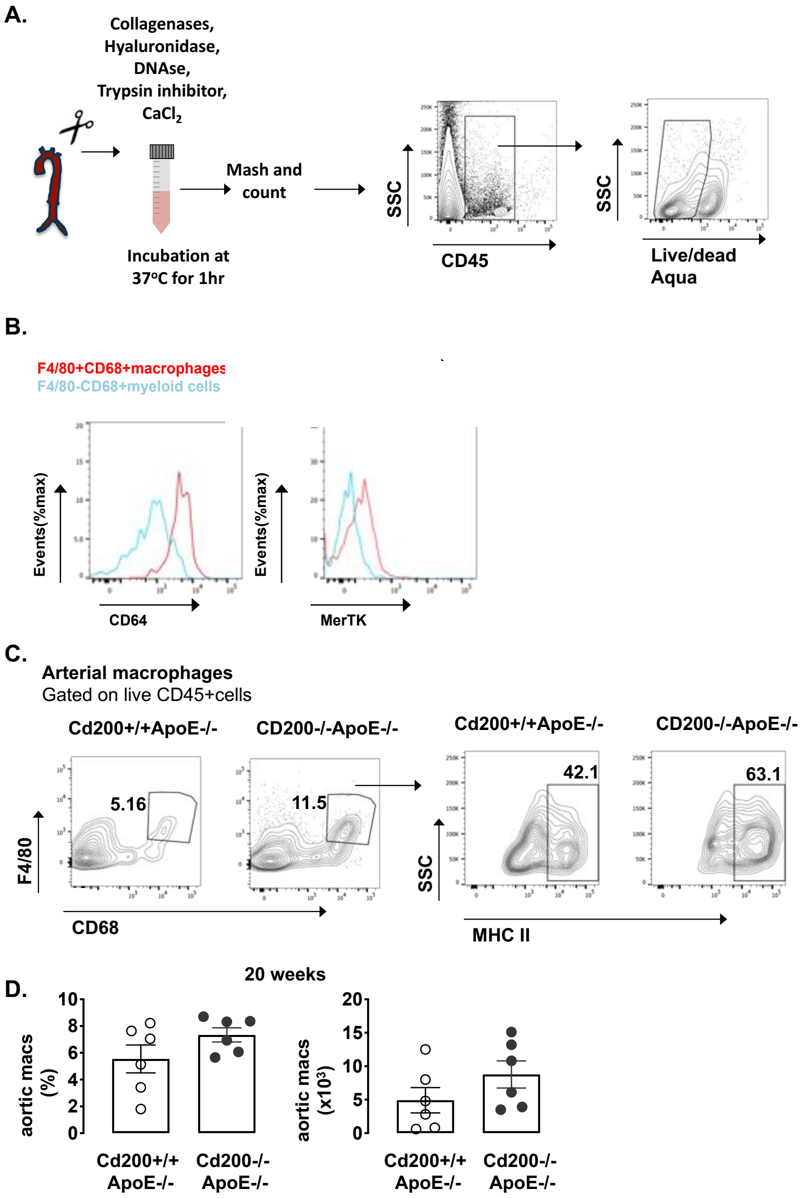

Online Figure V. Analysis of CD200+/+ApoE-/- and CD200-/-ApoE-/- mouse aortas by flow cytometry. (A) Gating strategy for live aortic CD45+ cells by flow cytometry. (B) Representative plots showing expression of CD64 and MerTK in F4/80+CD68+ cells. Plots that best represent the groups are shown (C) Representative contour plots show arterial F4/80+CD68+cells (gated on live CD45+ cells) and arterial major histocompatibility complex class II (MHCII)+ cells (gated on F4/80+CD68+ cells) in *Cd200+/+ApoE-/-* and *Cd200-/-ApoE-/-* mice. Plots that best represent the groups are shown (D) Graphs show the numbers (expressed as percentages and absolute numbers) of arterial F4/80+CD68+ cells in 20-week *Cd200+/+ApoE-/-* and *Cd200-/-ApoE-/-* mice (n=6). Bars denote group mean ± SEM.

Online Figure VI

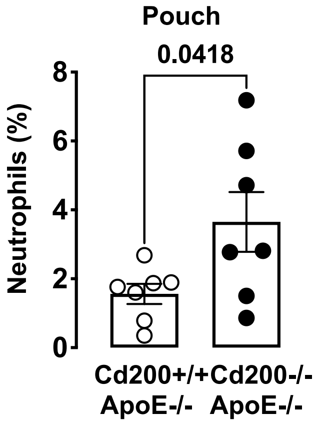

Online Figure VI. Numbers of neutrophils in the air pouch membrane of CD200+/+ApoE-/- and CD200-/-ApoE-/- mice. Graph shows the percentage of neutrophils (gated on live CD45+CD11b+F4/80-Ly6G+cells) out of singlets in 15 week old *Cd200+/+ApoE-/-* and *Cd200-/-ApoE-/-* mice (n=7) in the air pouch membrane. Bars denote group mean ± SEM.

Online Figure VII

A.

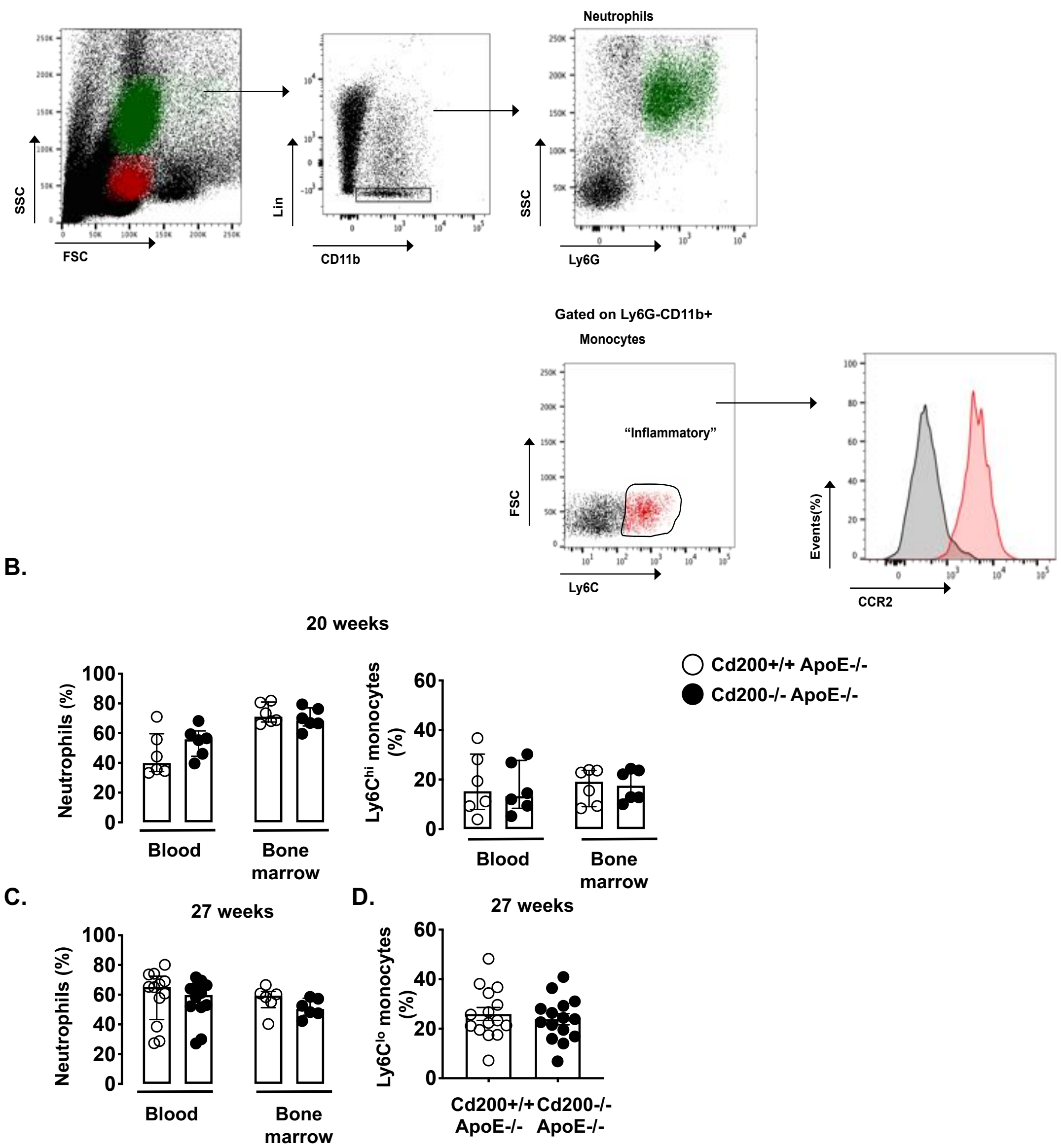

Online Figure VII. Analysis of the blood and bone marrow (BM) of CD200+/+ApoE-/- and CD200-/-ApoE-/- mice. (A) Gating strategy for blood monocytes and neutrophils in *Cd200+/+ApoE-/-* mice. (B) Percentage of neutrophils and monocytes in the blood and BM of 20-week old *Cd200+/+ApoE-/-* (open circles) and *Cd200-/-ApoE-/-* (closed circles) mice on chow diet (n=6). (C) Percentage of neutrophils in the blood and BM of 27-week old Cd200+/+*ApoE-/-* (open circles) and *Cd200-/-ApoE-/-* (closed circles) mice on chow diet (n=6-12). Bars denote group median ± interquartile range. (D) Percentage of Ly6C^{lo} monocytes in the blood of 27-week old *Cd200+/+ApoE-/-* and *Cd200-/-ApoE-/-* mice on chow diet (n=15). Bars denote group mean ± SEM.

Online Figure VIII

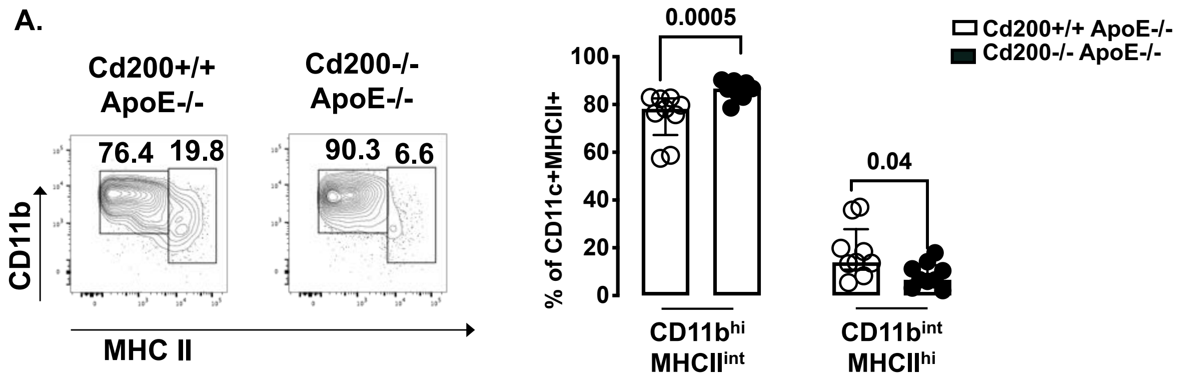

Online Figure VIII. Analysis of granulocyte-macrophage colony-stimulating factor (GM-CSF) bone marrow (BM)-derived cells from *Cd200+/+ApoE-/-* and *Cd200-/-ApoE-/-* mice. (A) Contour plots and graph show the percentage (%) of CD11b^{hi}MHCII^{int} macrophages and CD11b^{int}MHCII^{hi} DCs in GM-CSF-derived BM cultures in 27-week old *Cd200+/+ApoE-/-* and *Cd200-/-ApoE-/-* mice (n=9). Bars denote group median ± interquartile range.

Online Figure IX

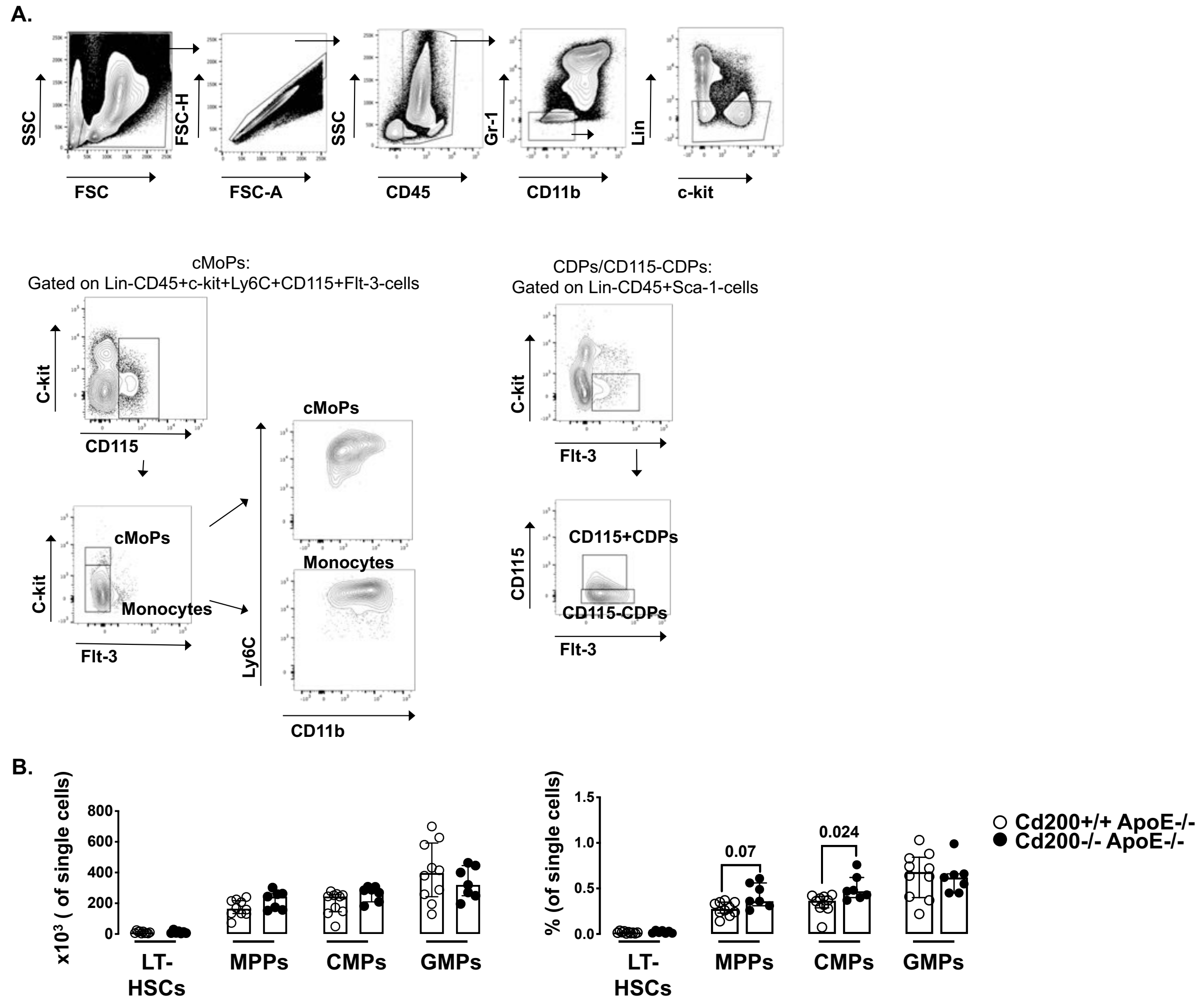

Online Figure IX. Gating strategy for haematopoietic stem cells and progenitor cells in the bone marrow (A) Gating strategy for bone marrow HSCs. Single cells and debris were excluded and cells were gated on Lin-CD45+c-kit+ cells. Lin−Sca-1+c-Kit+ (LSK) cells were defined as Sca-1+ckit^{hi}, multipotent progenitors (MPPs) as Sca-1+c-kit^{hi} CD34+CD150-, long term (LT)-HSCs as Sca-1+c-kit^{hi} CD34-CD150+, granulocyte-monocyte progenitors (GMPs) as Sca-1-c-kit^{hi} CD34+CD16/32+ and common myeloid progenitors (CMPs) as Sca-1-c-kit^{hi}CD34+CD16/32-. common monocyte progenitors (cMoPs) were gated as Lin-CD45+c-kit+Ly6C+CD115+Flt-3-cells and common dendritic cell progenitors CDPs were gated as Lin-CD45+Sca-1-cells. (B) Graphs show the numbers of c-kit+Sca-1+CD150+CD34-long term hematopoietic stem cells (LT-HSCs), c-kit+Sca-1+CD150-CD34+ multipotent progenitors (MPPs), c-kit+sca-1-CD16/32+CD34+ granulocyte-monocyte progenitors (GMPs), and c-kit+sca-1-CD16/32-CD34+ common myeloid progenitors (CMPs) in the bone marrow of 27-week old *Cd200+/+ApoE-/-* and *Cd200-/-ApoE-/-* mice (n=7-10). Bars denote group median ± interquartile range

Online Figure X

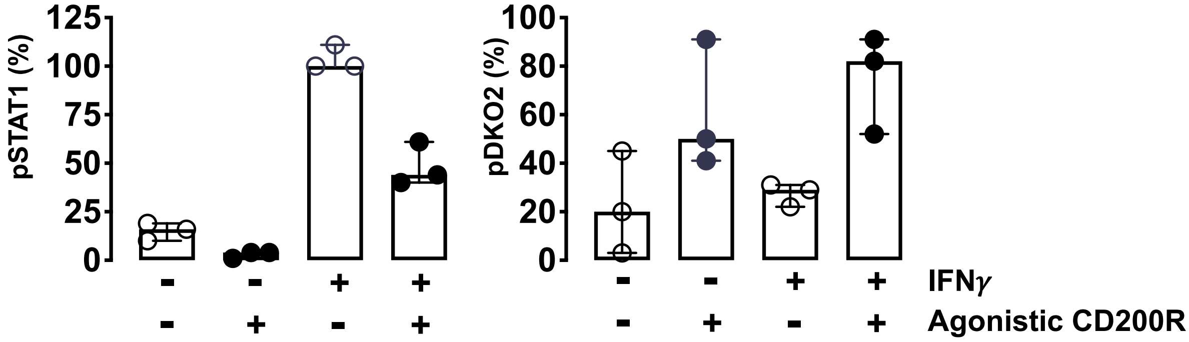

Online Figure X. CD200R agonism inhibits STAT1 phosphorylation in myeloid cells. Western blot analysis of pSTAT1, STAT1, pDOK2, DOK2 and clathrin heavy chain (CHC) in a macrophage cell line (RAW 64.7) upon stimulation with IFNγ and an agonistic CD200R antibody. Graphs show the level of STAT1 and DOK2 phosphorylation. Three individual experiments were performed. Bars show median ± interquartile range.

Online Figure XI

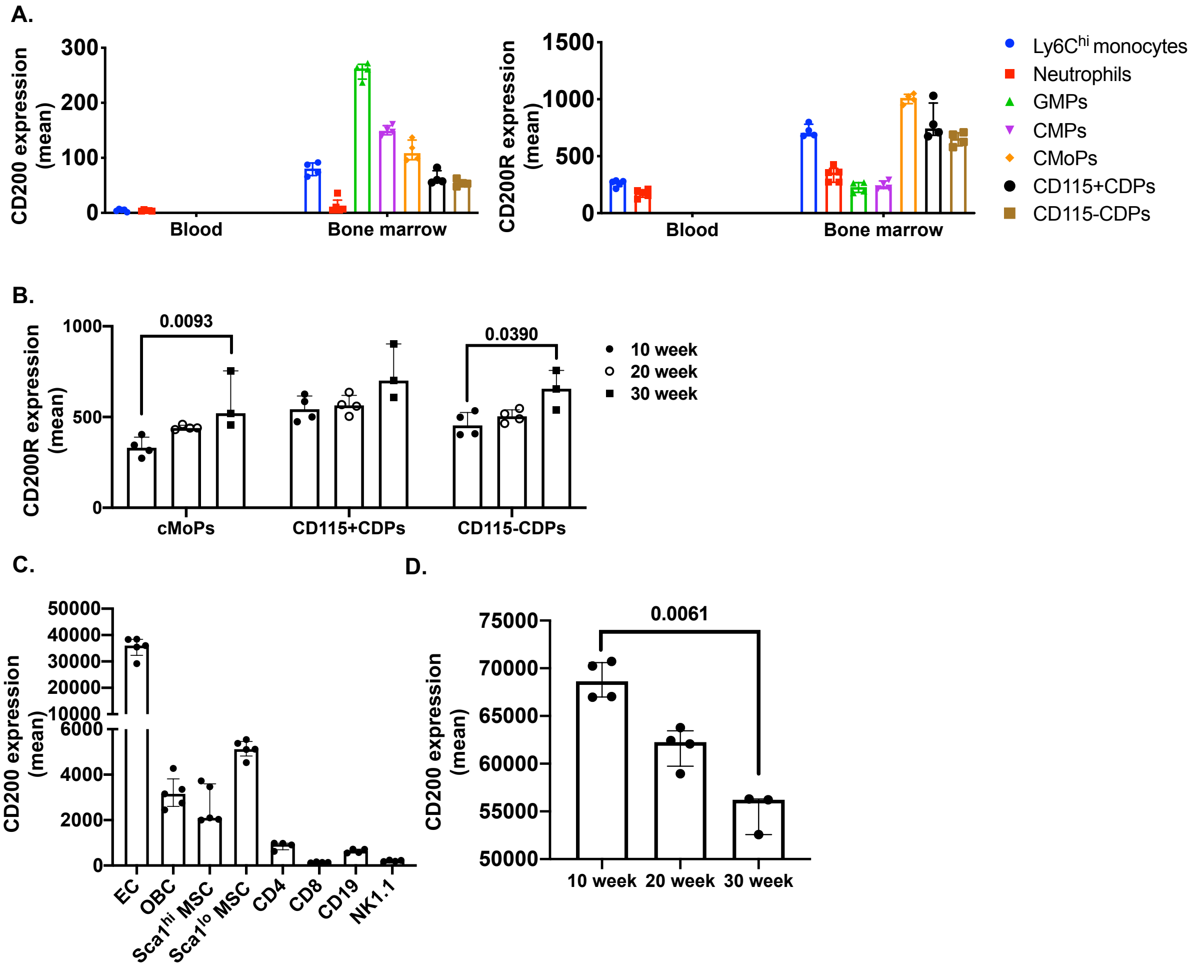

Online Figure XI. CD200 and CD200R expression in the blood and bone marrow of apolipoprotein E-deficient (ApoE-/-) mice. (A) Graphs show mean CD200 (left graph) and CD200R (right graph) expression in blood cells and in bone marrow progenitors of ApoE-/- mice by flow cytometry. (B) Graph shows mean CD200R expression on cMoPs and CDPs in the bone marrow of ApoE-/- aged 10, 20 and 30 weeks (C) Graph shows mean CD200 expression in bone marrow stromal and lymphoid cells of ApoE-/- mice. (D) Graph shows mean CD200 expression on bone marrow endothelial cells of ApoE-/- aged 10, 20 and 30 weeks. Bars denote group median ± interquartile range (n=3-5).

Online Figure XII

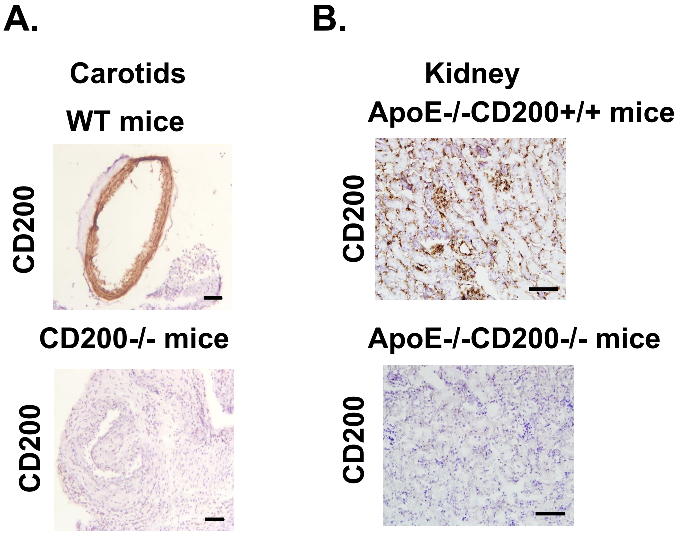

Online Figure XII. Validation of an anti-mouse CD200 antibody. (A) Representative images of carotid sections from wild type (WT) and *Cd200-/-* mice stained with an antibody against CD200 and haematoxylin. (B) Representative images of kidney sections from *CD200+/+ApoE-/-* and *Cd200-/-ApoE-/-* mice stained with an antibody against CD200 and haematoxylin. Images that best represent the staining observed are shown. scale bars = 100 μm
