# Supplementary figures and images for: CD200 Limits Monopoiesis and Monocyte Recruitment in Atherosclerosis
Source: Circ Res. 2021 May 12;129(2):280–95. doi: 10.1161/CIRCRESAHA.119.316062 (PMC8260471; doi:10.1161/CIRCRESAHA.119.316062)

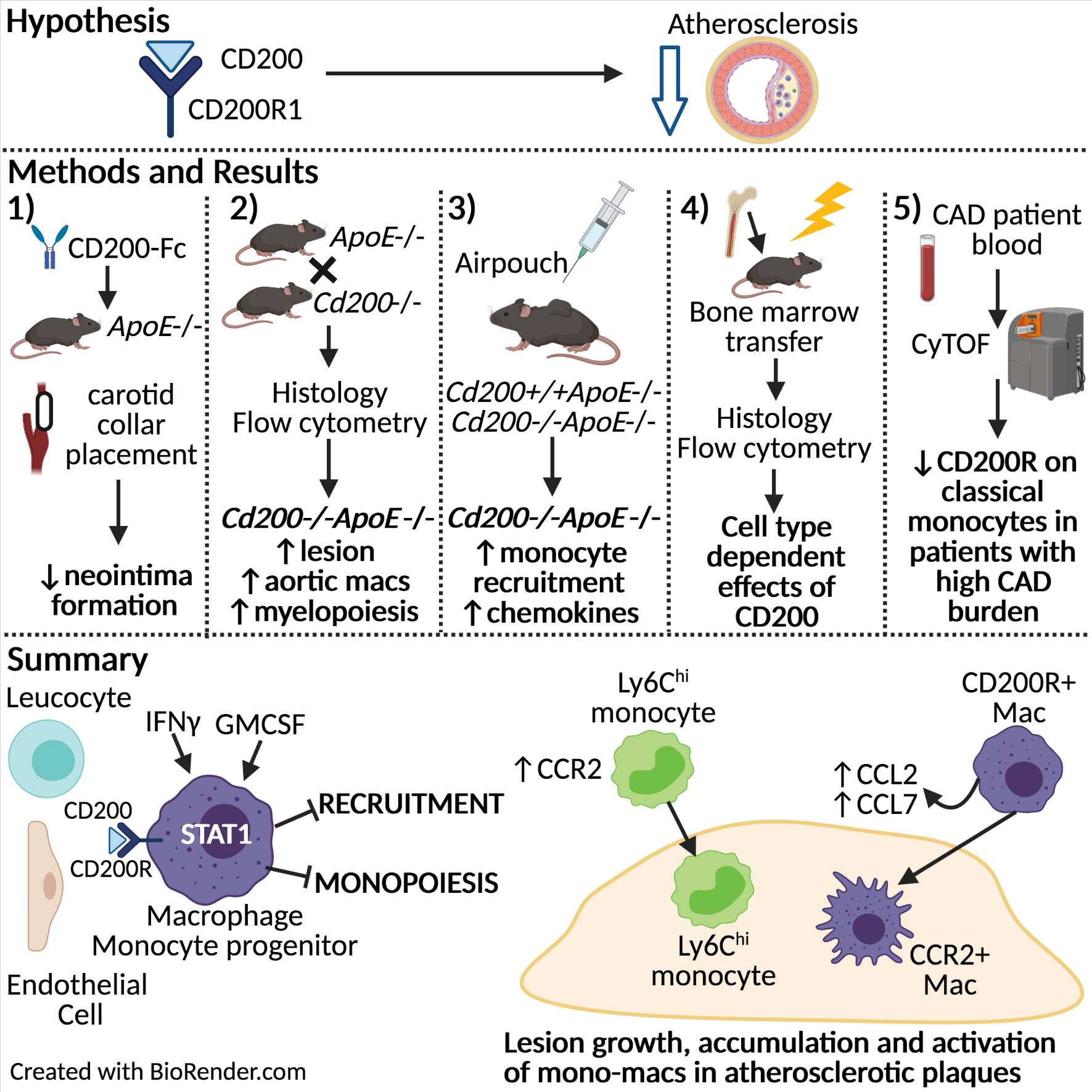

Supplement: Supplementary file 2 [file res-129-280-s002.jpg]
