# Supplementary material for: CD200 Limits Monopoiesis and Monocyte Recruitment in Atherosclerosis
Source: Circ Res. 2021 May 12;129(2):280–95. doi: 10.1161/CIRCRESAHA.119.316062 (PMC8260471; doi:10.1161/CIRCRESAHA.119.316062)

Figure 6B

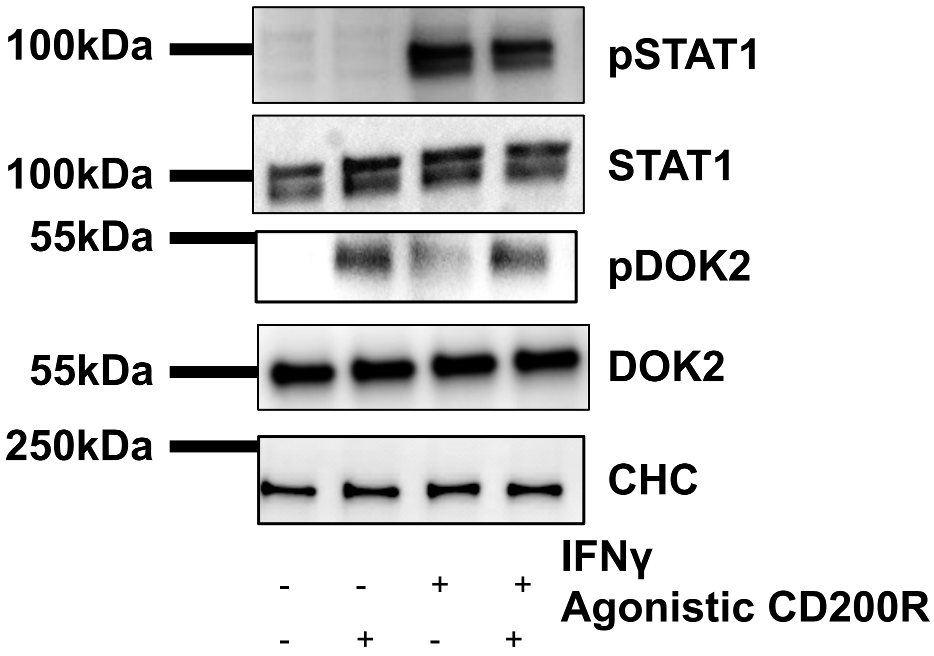

100kDa
pSTAT1
100kDa
STAT1
55kDa
pDOK2
55kDa
DOK2
250kDa
CHC
IFNγ
- - + +
Agonistic CD200R
- + - +

Figure 6B – Full blot pSTAT1

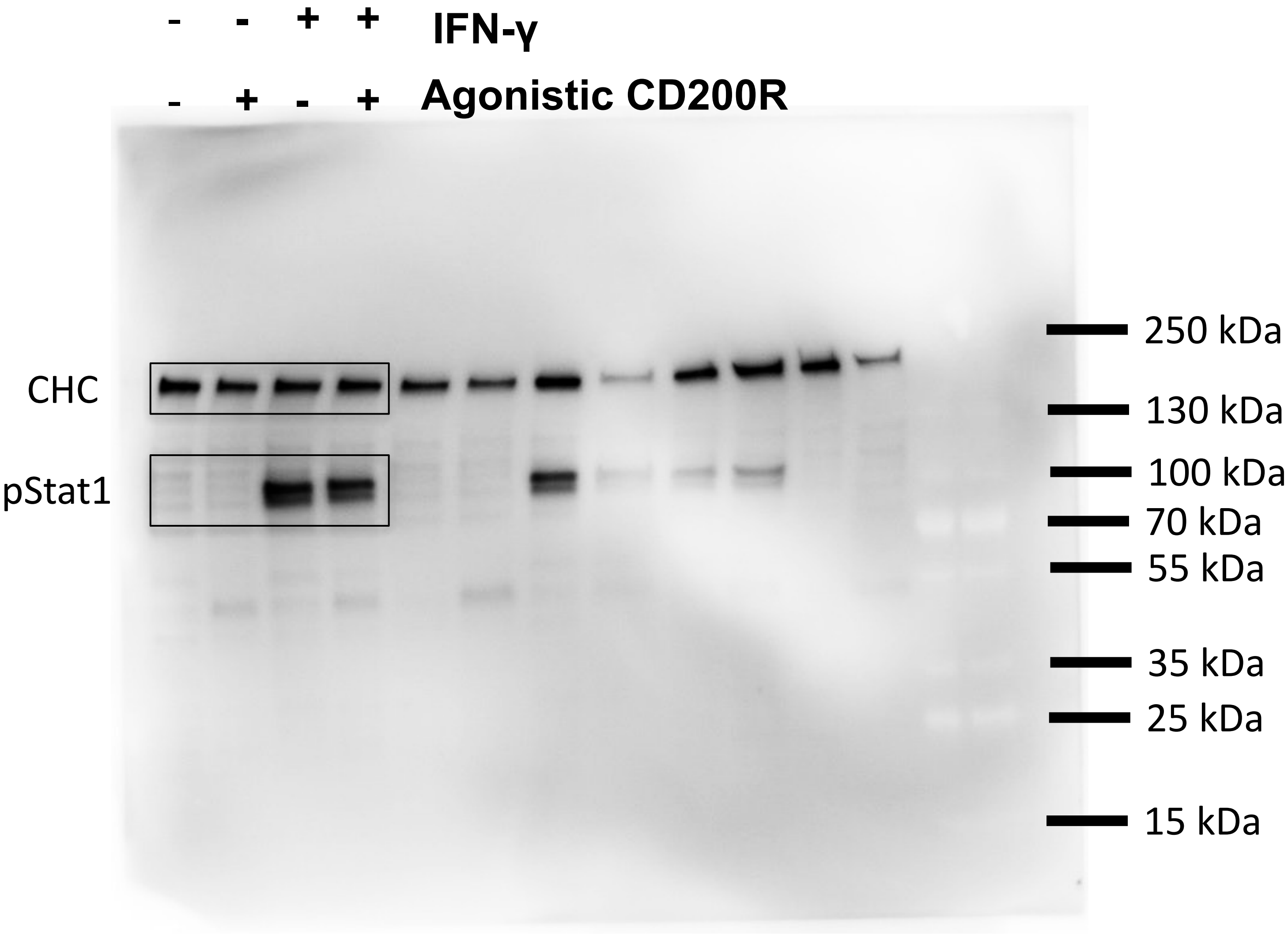

Figure 6B – Full blot STAT1

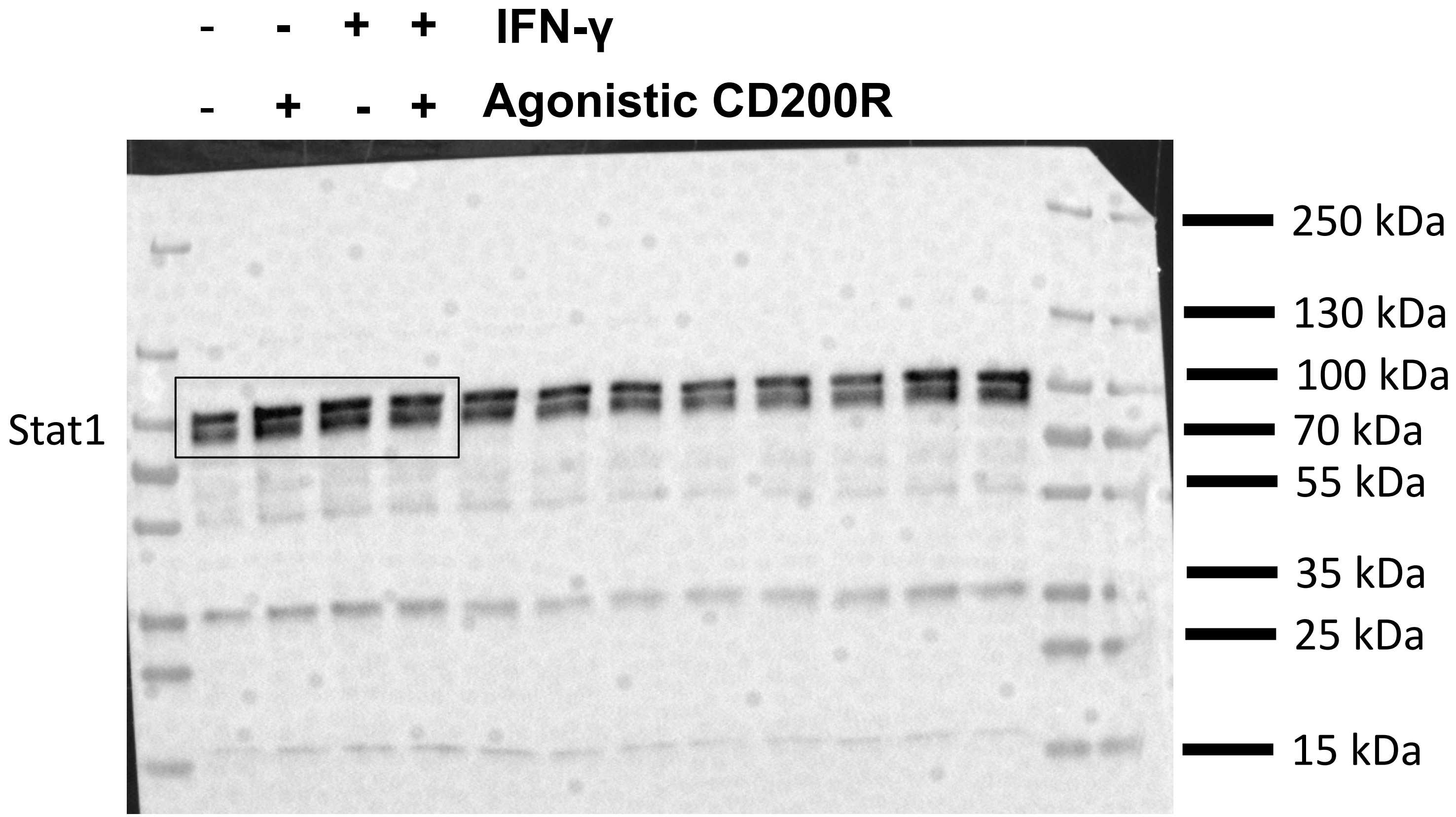

Figure 6B – Full blot pDKO2

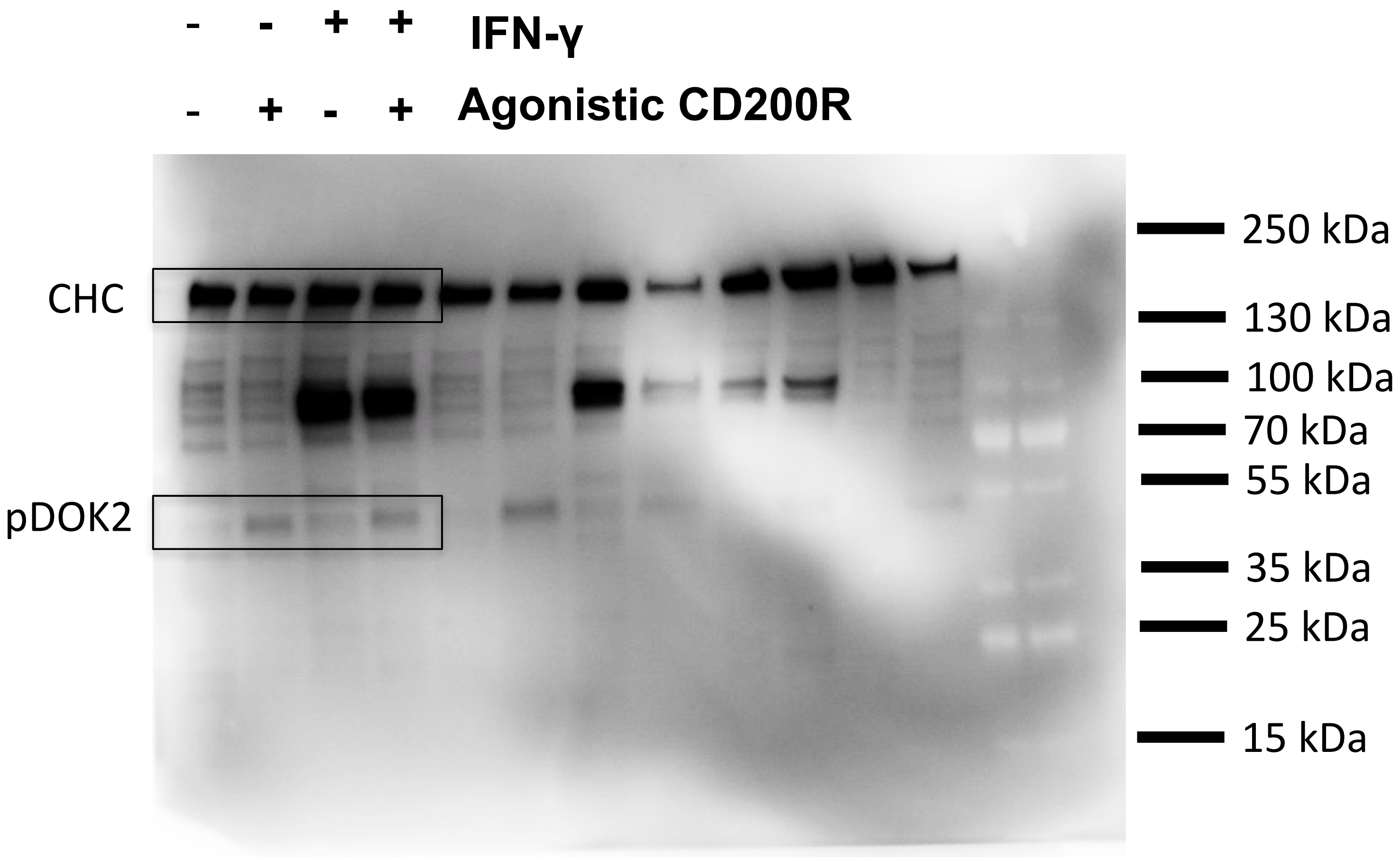

Figure 6B – Full blot DKO2

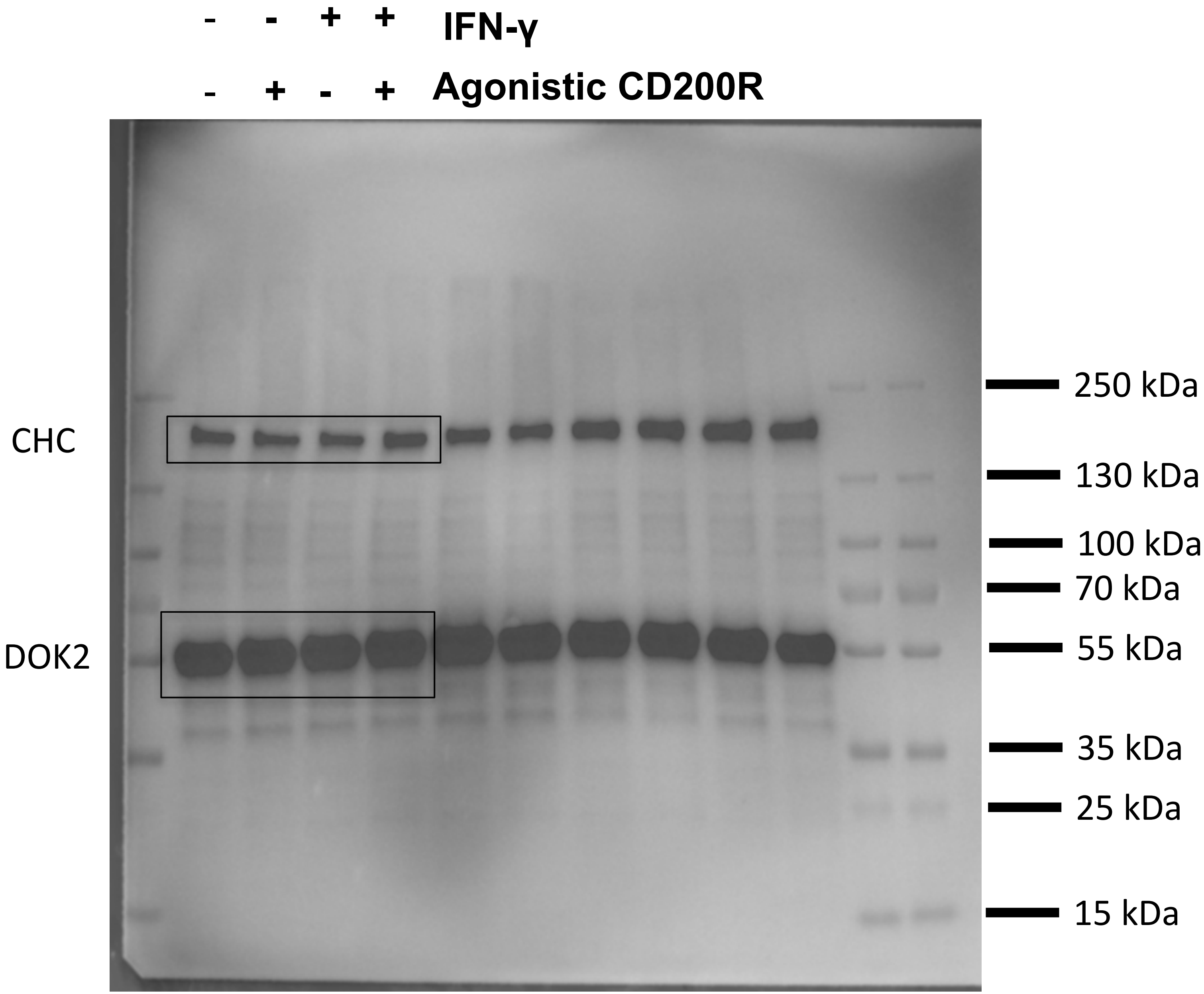

Supplement: Supplementary file 3 [file res-129-280-s003.pdf]
