# Supplementary material for: CD200 Limits Monopoiesis and Monocyte Recruitment in Atherosclerosis
Source: Circ Res. 2021 May 12;129(2):280–95. doi: 10.1161/CIRCRESAHA.119.316062 (PMC8260471; doi:10.1161/CIRCRESAHA.119.316062)
Supplement: Supplementary file 4 [file res-129-280-s004.pdf]

Major Resources Table

In order to allow validation and replication of experiments, all essential research materials listed in the Methods should be included in the Major Resources Table below. Authors are encouraged to use public repositories for protocols, data, code, and other materials and provide persistent identifiers and/or links to repositories when available. Authors may add or delete rows as needed.

Animals (in vivo studies)

Species	Vendor or Source	Background Strain	Sex	Persistent ID / URL
Mouse	Bred in House	C57BL/6J	M	

Genetically Modified Animals

	Species	Vendor or Source	Background Strain	Other Information	Persistent ID / URL
**Parent - Male**					
**Parent - Female**					

Antibodies

Target antigen	Vendor or Source	Catalog #	Working concentration	Lot # (preferred but not required)	Persistent ID / URL
B220 PerCP-Cy5.5	Biolegend	103236	1:200		
CCR2 PE	Biolegend	150610	1:50		
CCR2 APC	R&D systems	FAB5538A	0.2 µg/ml		
CD117 (c-kit) BV785	BD Biosciences	564012	1:100		
CD11b BV711	Biolegend	101242	1:200		
CD11b PE	eBioscience	12-0112-81	1:20		
CD11b PerCP	Biolegend	101230	1:100		
CD11b PerCP-Cy5.5	Biolegend	101228	1:100		
CD11c PerCP-Cy5.5	Biolegend	117328	1:100		
CD11c APC-Cy7	Biolegend	117324	1:200		
CD115 PE	Biolegend	135506	1:100		
CD115 APC	Biolegend	135510	1:100		
CD135 (Flt3) APC	Biolegend	135310	1:50		
CD135 (Flt3) PE	Biolegend	135306	1:50		
CD150 BV605	Biolegend	115927	1:200		
CD16/32 APC-Cy7	BD Biosciences	560541	1:100		
CD19 PerCP	Biolegend	115532	1:200		
CD200	AbD Serotec	MCA1958	5ug/ml		
CD200 APC	Biolegend	123810	1:50		
CD200R	AbD Serotec	MCA2281	10ug/ml		
CD200R PE	Biolegend	123908	1:50		
CD200R APC	Biolegend	123916	1:75		
CD200R Fitc	Biolegend	123910	1:50		
CD206 APC	Biolegend	141708	1:100		
CD206 Biotin	AbD Serotec	MCA2235B	1:200		
CD3 FITC	BD Bioscience	553062	1:100		
CD3 PerCP-Cy5.5	BD Bioscience	551183	1:100		
CD31 (PECAM-1)	Abcam	ab28364	0.04ug/ml		
CD31 (PECAM-1) BV605	Biolegend	102427	1:100		

DOI [to be added]

CD31 (PECAM-1) APC-Fire 750	Biolegend	102434	1:100		
CD34 FITC	eBiosciences	11-031-82	1:75		
CD4 BV421	Biolegend	100543	1:100		
CD4 PerCP-Cy5.5	eBioscience	45-0042-82	1:200		
CD45 Alexa700	Biolegend	103128	1:200		
CD45 APC	Biolegend	103111	1:200		
CD45 APC-Cy7	Biolegend	103116	1:200		
CD45 BV421	Biolegend	103134	1:200		
CD45 BV605	Biolegend	103139	1:50		
CD45 BV711	Biolegend	103147	1:200		
CD45 FITC	Biolegend	103108	1:500		
CD45 PE	Biolegend	103106	1:200		
CD45 PECy7	Biolegend	103113	1:200		
CD45 PerCP	Biolegend	103130	1:200		
CD45 PerCP-Cy5.5	Biolegend	103132	1:200		
CD64 APC	Biolegend	139306	1:20		
CD68	AbD Serotec	MCA1957GA	1:200		
CD68 PerCp Cy5.5	Biolegend	137010	1:200		
CD68 PECy7	Biolegend	137016	1:100		
CD68-AF647	Abd Serotec	MCA1957A647	1:200		
CD8 BV711	Biolegend	100759	1:100		
F4/80 BV605	Biolegend	123133	1:50		
F4/80 PE	Biolegend	123110	1:50		
FcεRIα PerCP-Cy5.5	Biolegend	134320	1:200		
Isotype control (Rabbit IgG)	Abcam	ab37415	As for antibody of interest		
Isotype control (Rat IgG1)	AbD Serotec	MCA6004GA	As for antibody of interest		
Isotype control (Rat IgG2α κ)	AbD Serotec	MCA1212	As for antibody of interest		
Ki-67 BV421	BD Bioscience	562899	1:50		
Ly6C PECy7	Biolegend	128018	1:50		
Ly6G APC	Biolegend	127614	1:200		
Ly-6G/C (Gr-1) PE	Biolegend	108408	1:250		
MerTK PE	R&D systems	FAB5912P	1:50		
MHCII BV421	Biolegend	107632	1:100		
MHCII FITC	Biolegend	107606	1:100		
NKp46 PerCP-Cy5.5	Biolegend	137610	1:200		
PDGFRa PE-Dazzle	Biolegend	135921	1:100		
Sca-1 PerCp Cy5.5	Biolegend	108123	1:100		
Sca-1 Pacific Blue	Biolegend	108120	1:100		
SMC α-actin	Sigma Aldrich	A5691-.2ml	1:200		
TER-119 PE	Biolegend	116207	1:200		
TER-119 PerCP-Cy5.5	Biolegend	116228	1:200		
Ly-6G/C (Gr-1)	Fluidigm	3141005B	0.1μl per $3x10^6$ cells		
CD11c	Fluidigm	3142003B	0.5μl per $3x10^6$ cells		
IL7Ra	Biolegend	135029	2μg/mL		
XCR1	Biolegend	148202	1μg/mL		
TCRgd	Biolegend	118101	4μg/mL		

DOI [to be added]

CX3CR1	Biolegend	149002	0.5µg/mL		
CD45	Fluidigm	3147003B	0.1µl per 3x10^6 cells		
CD11b (Mac-1)	Fluidigm	3148003B	0.075µl per 3x10^6 cells		
CD19	Fluidigm	3149002B	0.4µl per 3x10^6 cells		
CD24	Fluidigm	3150009B	0.1µl per 3x10^6 cells		
CD64	Fluidigm	3151012B	0.5µl per 3x10^6 cells		
CD3e	Fluidigm	3152004B	0.8µl per 3x10^6 cells		
CD200R	Biolegend	123902	0.1µg/mL		
CD43	Biolegend	143202	0.125µg/mL		
Lyve-1	eBioscience	14-0443-95	0.5µg/mL		
CD169	Biolegend	142402	0.5µg/mL		
CD206	Biolegend	141702	0.1µg/mL		
F4/80	Fluidigm	3159009B	0.5µl per 3x10^6 cells		
CD26	Biolegend	137802	4µg/mL		
CD103	Biolegend	121402	4µg/mL		
Ly6C	Fluidigm	3162014B	0.1µl per 3x10^6 cells		
CCR2	Biolegend	Custom order	0.5µg/mL		
CD172a (SIRPa)	Biolegend	144002	1µg/mL		
CD161 (NK1.1)	Fluidigm	3165018B	1µl per 3x10^6 cells		
CD209b (SIGNR1)	Biolegend	147802	0.5µg/mL		
SIGLECF	BD	552125	0.125µg/mL		
CD8a	Fluidigm	3168003B	0.5µl per 3x10^6 cells		
TCRb	Fluidigm	3169002B	0.2µl per 3x10^6 cells		
CD90.2	Biolegend	105333	0.15µg/mL		
CD44	Fluidigm	3171003B	0.1µl per 3x10^6 cells		
CD4	Fluidigm	3172003B	0.4µl per 3x10^6 cells		
SIGLECH	Biolegend	129602	0.5µg/mL		
I-A/I-E (MHCII)	Fluidigm	3174003B	0.1µl per 3x10^6 cells		
CD68	Biolegend	137002	0.25µg/mL		
CD45R (B220)	Fluidigm	3176002B	0.5µl per 3x10^6 cells		
CD70	Biolegend		2µl per 3x10^6 cells		
CD19	Fluidigm	3142001B	0.03125µl per 3x10^6 cells		
CD123	Fluidigm	3143014B	0.25µl per 3x10^6 cells		
CD11b	Fluidigm	3144001B	0.5µl per 3x10^6 cells		
IgD	Fluidigm	3146005B	0.03125µl per 3x10^6 cells		
CD11c	Fluidigm	3147008B	0.0625µl per 3x10^6 cells		
PD-L1 (CD274)	Fluidigm	3148017B	0.5µl per 3x10^6 cells		
CD200	Fluidigm	3149007B	1µl per 3x10^6 cells		
CD43	Fluidigm	3150006B	0.25µl per 3x10^6 cells		
CD14	Fluidigm	3151009B	0.5µl per 3x10^6 cells		
CD95	Fluidigm	3152017B	1µl per 3x10^6 cells		
TIM-3	Fluidigm	3153008B	0.5µl per 3x10^6 cells		
BAFFR (CD268)	Biolegend	316902	0.5µl per 3x10^6 cells		
CD86	Fluidigm	3156008B	0.25µl per 3x10^6 cells		
CD137L	Fluidigm	3158022B	2µl per 3x10^6 cells		
CD22	Fluidigm	3159005B	0.25µl per 3x10^6 cells		
CD200R	Biolegend	329302	2.5µl per 3x10^6 cells		
CD80	Fluidigm	3162010B	1µl per 3x10^6 cells		
CD95L	Biolegend	306402	2µl per 3x10^6 cells		
R+D Systems	R+D Systems	MAB6942-100	3µl per 3x10^6 cells		

DOI [to be added]

CD40	Fluidigm	3165005B	0.5µl per $3x10^6$ cells		
Siglec 10	Biolegend		2µl per $3x10^6$ cells		
CD27	Fluidigm	3167006B	0.05µl per $3x10^6$ cells		
OX40L	R+D Systems	MAB10541-100	3µl per $3x10^6$ cells		
CD24	Fluidigm	3169004B	1µl per $3x10^6$ cells		
CD3	Fluidigm	3170001B	0.1µl per $3x10^6$ cells		
CD20	Fluidigm	3171012B	0.05µl per $3x10^6$ cells		
IgM	Fluidigm	3151026D	0.0625µl per $3x10^6$ cells		
CD137	Fluidigm	3173015B	2µl per $3x10^6$ cells		
HLA-DR	Fluidigm	3174001B	0.1µl per $3x10^6$ cells		
PD-1	Fluidigm	3175008B	0.5µl per $3x10^6$ cells		
CD56	Fluidigm	3176009B	1µl per $3x10^6$ cells		
CD16	Fluidigm	3209002B	1µl per $3x10^6$ cells		
CD45	Fluidigm	3089003B	1µl per $3x10^6$ cells		
pSTAT1	Cell signaling technologies	9167	1:1000		
STAT1	Cell signaling technologies	9172	1:1000		
pDOK2	Cell signaling technologies	3911S	1:1000		
DOK2	BD Bioscience	611974	1:1000		
Anti-mouse secondary	Sigma	A9044-2ml	1:10,000		
Anti-rabbit secondary	Sigma	A9169	1:10,000		
Anti-clathrin heavy chain	BD Bioscience	610500	1:5000		
Biotinylated anti-rat IgG	Vector laboratories	BA4001	1:400		
Streptavidin-488	ThermoFisher	S32354	1:500		
Streptavidin-568	ThermoFisher	S11226	1:500		
CD200R (OX131)	Absolute Antibody	Ab00111-1.1	2.5-10ug/ml		
CD200	abcam	ab203887	1:250		
CD200R	abcam	ab198010	1:100		
CD68	abcam	ab201340	1:300		
CD31	Dako/Agilent	M082301-2	1:20		
Smooth muscle actin	Sigma	F3777	1:3000		
Biotinylated anti-rabbit	Dako/Agilent	E0432	1:400		
Biotinylated anti-mouse	Jackson Immunoresearch		1:500		
Anti-Fitc	Jackson Immunoresearch		1:600		

DNA/cDNA Clones

Clone Name	Sequence	Source / Repository	Persistent ID / URL

Cultured Cells

DOI [to be added]

Name	Vendor or Source	Sex (F, M, or unknown)	Persistent ID / URL

Data & Code Availability

Description	Source / Repository	Persistent ID / URL

Other

Description	Source / Repository	Persistent ID / URL
Live/Dead Fixable Aqua	ThermoFisher	Catalogue Number L34957
Rhodium interchelator	Fluidigm	Catalogue Number 201103A
Iridium interchelator	Fluidigm	Catalogue Number 201192A
Mouse Fc block	BD Bioscience	Catalogue Number 553142
26-plex mouse procartaplex panel 1	ThermoFisher	Catalogue Number EPXR260-26088-901
Mm00441242_m1 for CCL2	ThermoFisher	Catalogue Number 4331182
Mm99999051_gh for CCR2	ThermoFisher	Catalogue Number 4331182
Mm01168134_m1 for IFNγ	ThermoFisher	Catalogue Number 4331182
Mm00487740_m1 for CD200	ThermoFisher	Catalogue Number 4331182
Mm00491164_m1 for CD200R1	ThermoFisher	Catalogue Number 4331182
Mm03047340_m1 for CD68	ThermoFisher	Catalogue Number 4331182
Mm01288993_m1 for IL-12β	ThermoFisher	Catalogue Number 4331182
JAK inhibitor CEP-3377P	Selleckchem	Catalogue Number 52806
Mouse biotin lineage antibody cocktail	ThermoFisher	Catalogue Number 88-7774-75
